# Genome Mining of Plant NPFs Reveals Varying Conservation of Signature Motifs Associated With the Mechanism of Transport

**DOI:** 10.3389/fpls.2018.01668

**Published:** 2018-12-04

**Authors:** Antonella Longo, Nicholas W. Miles, Rebecca Dickstein

**Affiliations:** ^1^BioDiscovery Institute, University of North Texas, Denton, TX, United States; ^2^Department of Biological Sciences, University of North Texas, Denton, TX, United States

**Keywords:** nitrate peptide family (NPF) transporters, nitrogen, proton-dependent oligopeptide transporter, genome, nitrate, phytohormones (auxin, gibberellin), glucosinolates (GSL)

## Abstract

Nitrogen is essential for all living species and may be taken up from the environment in different forms like nitrate or peptides. In plants, members of a transporter family named NPFs transport nitrate and peptides across biological membranes. NPFs are phylogenetically related to a family of peptide transporters (PTRs) or proton-coupled oligopeptide transporters (POTs) that are evolutionarily conserved in all organisms except in Archaea. POTs are present in low numbers in bacteria, algae and animals. NPFs have expanded in plants and evolved to transport a wide range of substrates including phytohormones and glucosinolates. Functional studies have shown that most NPFs, like POTs, operate as symporters with simultaneous inwardly directed movement of protons. Here we focus on four structural features of NPFs/POTs/PTRs that have been shown by structural and functional studies to be essential to proton-coupled symport transport. The first two features are implicated in proton binding and transport: a conserved motif named ExxER/K, located in the first transmembrane helix (TMH1) and a D/E residue in TMH7 that has been observed in some bacterial and algal transporters. The third and fourth features are two inter-helical salt bridges between residues on TMH1 and TMH7 or TMH4 and TMH10. To understand if the mechanism of transport is conserved in NPFs with the expansion to novel substrates, we collected NPFs sequences from 42 plant genomes. Sequence alignment revealed that the ExxER/K motif is not strictly conserved and its conservation level is different in the NPF subfamilies. The proton binding site on TMH7 is missing in all NPFs with the exception of two NPFs from moss. The two moss NPFs also have a positively charged amino acid on TMH1 that can form the salt bridge with the TMH7 negative residue. None of the other NPFs we examined harbor residues that can form the TMH1–TMH7 salt bridge. In contrast, the amino acids required to form the TMH4–TMH10 salt bridge are highly conserved in NPFs, with some exceptions. These results support the need for further biochemical and structural studies of individual NPFs for a better understanding of the transport mechanism in this family of transporters.

## Introduction

The first proton-dependent nitrate transporter in plants was isolated in *Arabidopsis thaliana* from a mutant that conferred resistance to the herbicide chlorate and resulted in a decreased nitrate uptake (Tsay et al., 1993). At the time of its discovery, the new protein, initially called CHL1 (later NRT1.1 and now AtNPF6.3), did not have sequence similarity with any known protein. Later, when peptide transport proteins from bacteria and animals were isolated, sequence alignment with AtNPF6.3 supported the idea that they all belonged to a new family of transporters. The newly discovered proteins were classified as POTs (for Proton-dependent Oligopeptide Transporters) (Paulsen and Skurray, 1994) or the more generic designation of PTRs (for Peptide Transporters) (Steiner et al., 1995). In algae, the phylogenetically related nitrate transporters are known as NRT1s (Sanz-Luque et al., 2015). In 2014, a phylogenetically based nomenclature was proposed for the NRT1/PTRs transporters in plants, resulting in their renaming as NPFs (for NRT1/PTR Family) (Léran et al., 2014). NPFs were classified into eight different subfamilies, NPF1 to 8, with members of each family assigned an increasing number resulting in a two number code for each NPF (Léran et al., 2014). Thus, *A. thaliana* CHL1/NRT1.1 was renamed AtNPF6.3. NPF/POT/PTR transporters are part of the major facilitator superfamily of secondary active transporters and designated as belonging to group 2.A.17 in the Transporter Classification Database (TCDB) (Saier et al., 2016).

In bacteria and animals POT/PTR transporters are present in low numbers (one to four in bacteria; one in yeast; three in *Drosophila* and *Caenorhabditis elegans*; four in humans). In algae, NRT1s are present in one or two copies, with some algae having none at all. In contrast, plant genomes contain a much larger number of NPFs, from as low as 20 members in the moss *Physcomitrella patens* to as high as 115 in *Glycine max* (Léran et al., 2014). The high number of NPFs in plants is not surprising as plant genomes have an abundance of duplicated genes due both to whole genome and single-gene duplication events, with an average of 65% of genes with a duplicate copy (Panchy et al., 2016). Since retention of duplicated genes has been linked to the acquisition of new functions, the large number of NPF paralogs retained in plant genomes suggests that these transporters have evolved to play new essential roles in plants with non-overlapping functions. Indeed, NPFs have been shown to transport many different substrates, including glucosinolates and phytohormones like auxin, gibberellin and jasmonates. Most NPFs can transport more than one substance, but never peptides and nitrate (Léran et al., 2014; Corratgé-Faillie and Lacombe, 2017).

Even though a direct correlation between NPF sequences and substrate specificity has not been established yet, the transport mechanism is expected to be conserved independently from the substrate transported. Functional studies have shown that most NPFs power and coordinate the inward uptake of the substrate with an inwardly directed influx of protons. Therefore, NPFs function as proton-substrate symporters, similarly to the evolutionary related bacterial and animal POTs. Yet, there are exceptions: some NPFs mediate passive nitrate or chloride efflux in roots with no simultaneous transport of protons (Segonzac et al., 2007; Taochy et al., 2015; Li et al., 2016; Li B. et al., 2017); others are bi-directional transporters (Lin et al., 2008; Léran et al., 2013); others have been shown to be proton-coupled potassium antiporters (Li H. et al., 2017). These examples show the unique versatility of NPFs that is not limited to the ability of transporting a variety of different substrates, but also extends to include different modality of functioning.

**FIGURE 1:**
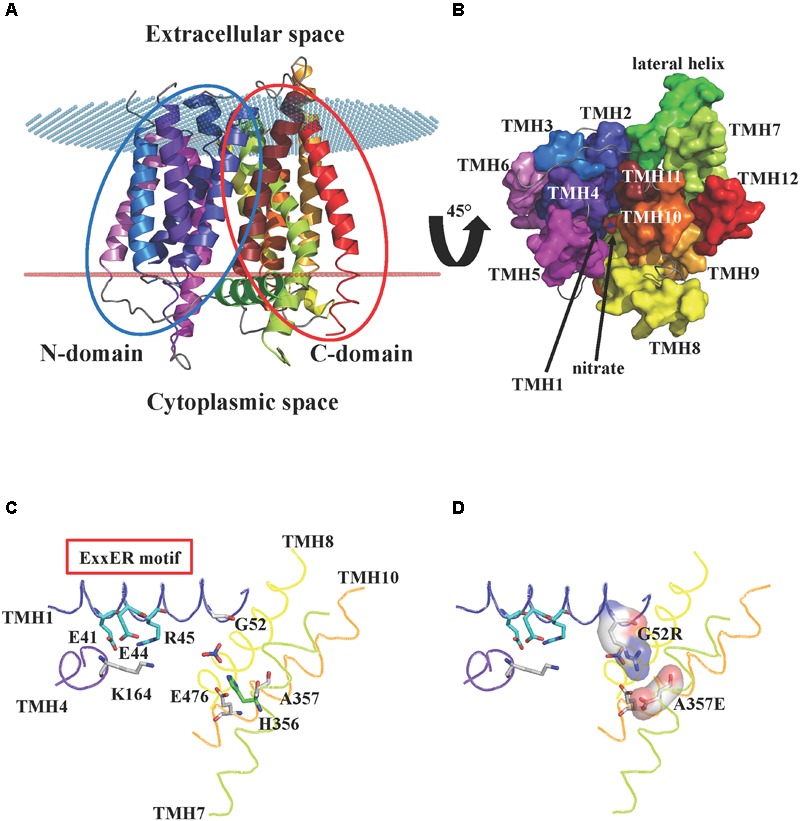
Crystal structure of AtNPF6.3. **(A)** Cartoon representation of the *A. thaliana* dual affinity nitrate transporter AtNPF6.3/NRT1.1 (PDB: 4OH3) (Sun et al., 2014) showing the core 12 transmembrane helices (TMHs) arranged into N- and C-terminal bundles (*blue* and *red ovals*, respectively). The transporter is in an inward-open conformation with the opening facing the cytoplasm. Spatial position of AtNPF6.3 in the membrane was calculated using the PPM server (Lomize et al., 2012): membrane boundaries are shown as *red* and *blue dots*. **(B)** AtNPF6.3 structure as seen from the cytoplasmic side. Nitrate is located at the bottom of the substrate channel. **(C)** Close-in view of the substrate channel formed by TMH1, 4, 7, and 10. Important side chains are shown as sticks: Glu41, Glu44 and Arg45 belong to the ExxER/K motif; Lys164 and Glu476 can potentially form an inter-helical salt bridge between TMH4 and TMH10 in the outward open conformation; residues Gly52 and Ala357 correspond to residues that have been shown to form a salt bridge between TMH1 and TMH7 in some bacterial and algal POTs/NRT1s. **(D)** *In silico* mutagenesis shows that when residues Gly52 and Ala357 are mutated into arginine and glutamate, respectively, they are within hydrogen bond distance and could form a salt bridge stabilizing the inward-open conformation.

NPFs are integral membrane proteins predicted to contain twelve transmembrane domains, with both N- and C-termini located at the cytosolic membrane side. So far, the lone crystal structure for a plant NPF is that of AtNPF6.3, the dual-affinity nitrate transporter that also transports auxin and functions as a nitrate sensor or transceptor (Ho et al., 2009; Krouk et al., 2010; Gojon et al., 2011). The AtNPF6.3 structure was independently solved by two groups in 2014, Parker and Newstead (2014) and Sun et al. (2014). The structures revealed a conserved topology of twelve transmembrane α-helices (TMHs) organized into two domains: an N-terminal domain that includes TMH1-6, and a C-terminal domain with TMH7-12 (Figure 1A). The two domains or bundles, are related by a pseudo twofold symmetry axis perpendicular to the membrane plane. Four transmembrane helices from each bundle (TMH1, 2, 4, and 5 from the N-terminal domain, and TMH7, 8, 10 and 11 from the C-terminal one) form a hydrophilic cavity that is responsible for proton and substrate binding. The remaining TMHs (TMH3, 6, 9, and 12) do not contribute to the core of the transporter, but have roles as scaffold. The AtNPF6.3 structure also includes a hydrophilic region located in the cytoplasm, between the N- and the C-domains: this region has been named lateral helix and its function is unknown (Figure 1B). AtNPF6.3 structures were solved in the inward-open conformation (open toward the intracellular space or cytoplasm) as apo-protein (Parker and Newstead, 2014) or in complex with nitrate (Parker and Newstead, 2014; Sun et al., 2014). The nitrate is located at the bottom of the internal cavity, close to His356 (Figure 1B).

**FIGURE 2:**
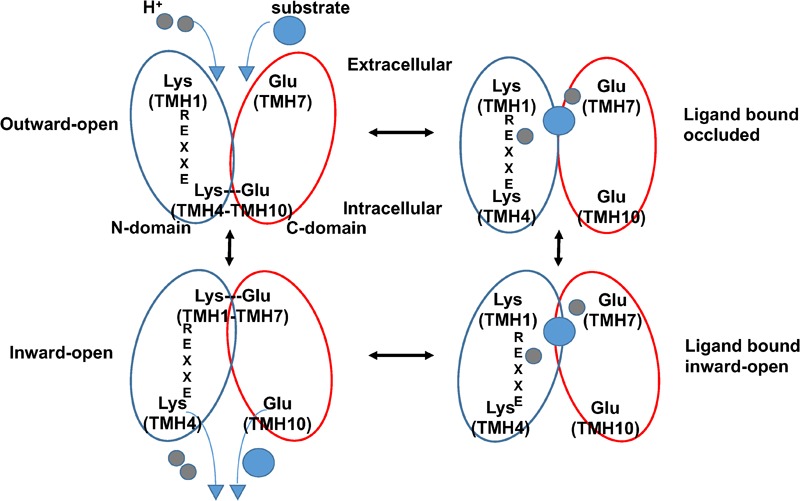
Proposed alternating-access mechanism in NPF/POT transporters. Schematic model of the proposed NPF/POT alternating-access transport cycle showing four conformations (based on Newstead, 2015). In the outward-open conformation, the transporter is open towards the extracellular space; a salt bridge forms between residues on TMH4 and TMH10, holding the N- and C-terminal bundles (*blue* and *red ovals*, respectively) together and stabilizing the conformation. Negatively charged amino acids in TMH7 and in the ExxER/K motif on TMH1 are exposed in the internal cavity and are available for proton binding. After protons (*gray sphere*s) and substrate (*blue sphere*) bind, the protein undergoes conformational changes that include the disruption of the TMH4–TMH10 salt bridge allowing the transporter to open towards the intracellular space in the inward-open conformation. Finally, protons and substrate are released in the cytoplasm. A salt bridge may form in the inward-open conformation between oppositely charged residues on TMH1 and TMH7 as observed in some crystal structures from bacterial POTs.

The crystal structures of AtNPF6.3 together with those of several bacterial POTs (Table 1), in combination with modeling, molecular dynamics, and functional assays, have helped advance our understanding of how this family of transporters couples proton intake with substrate transport (Newstead, 2011, 2015, 2017; Fowler et al., 2015). In the current model, transporters undergo structural changes in three major steps from outward to occluded to inward facing conformations, resulting in an alternate access cycle during which the ligand binding site is alternatively exposed to either side of the membrane (Figure 2). The cycle involves the following: (1) in the outward-open conformation, protons bind to chargeable amino acids in the interior of the hydrophilic substrate-binding cavity open toward the exterior followed by substrate binding to a residue located at the bottom of the cavity; (2) helical rearrangements break a salt bridge located toward the cytoplasmic side of the transporter; (3) the transporter switches to the inward-open conformation with substrate and protons released in the cytoplasm (Figure 2).

**Table 1:** List of available crystal structures for NPF/POT transporters.

Protein	Organism	ExxER motif	TMH4–TMH10 Salt bridge	TMH1–TMH7 Salt bridge	Conformation	Ligand	PDB ID	Reference
AtNPF6.3	*Arabidopsis thaliana*	**E**AV**ER**	Yes	No	Inward-open Inward-open Inward-open	None Nitrate Nitrate	5A2N 5A2O 4OH3	Parker and Newstead, 2014 Sun et al., 2014
PepT_So_	*Shewanella oneidensis*	**E**AC**ER**	Yes	Yes	Occluded state Inward-open	None None	2XUT 4UVM	Newstead et al., 2011 Fowler et al., 2015
PepT_So2_	*Shewanella oneidensis*	**E**LW**ER**	Yes	No	Inward-open Inward-open Inward-open Inward-open	Alafosfalin Ala-Ala-Ala Ala-Tyr-(Br) Ala-Tyr-(Br)-Ala	4LEP 4TPJ 4TPH 4TPG	Guettou et al., 2013 Guettou et al., 2014
PepT_St_	*Streptococcus thermophilus*	**E**MW**ER**	Yes	Yes	Inward-open Inward-open Inward-open	None None None	4APS 4D2B 5MMT	Solcan et al., 2012 Lyons et al., 2014 Quistgaard et al., 2017
GkPOT	*Geobacillus kaustophilus*	**E**FW**ER**	Yes	No	Inward-open Inward-open	None Alafosfalin	4IKV 4IKZ	Doki et al., 2013
YbgH	*Escherichia coli*	QIW**E**Y	Yes	No	Inward-open	None	4Q65	Zhao et al., 2014
YePEPT	*Yersinia enterocolitica*	**E**MW**ER**	Yes	Yes	Inward-open	None	4W6V	Boggavarapu et al., 2015
PepT_Xc_	*Xanthomonas campestris*	**E**AC**ER**	Yes	Yes	Auto-inhibited	None	6EI3	Parker et al., 2017

*The sole crystal structure of an NPF transporter, NPF6.3 from *A. thaliana* was obtained by two different groups and solved as an apo-protein or in the presence of nitrate. The crystal structures of seven POT proteins from six different bacteria were solved as apo-proteins, in complex with the peptide mimic alafosfalin or with di- or tri-peptides. Protein Data Bank Identification Codes (PDB ID) for each solved structure are included.*

Comparison between known structures indicates that residues required for coupling proton movement to structural changes during transport are conserved in these transporters. In particular, four structural features have been implicated in the current model: a conserved proton binding sequence called the ExxER/K motif on TMH1, a proton binding glutamate/aspartate residue on TMH7, and two sets of inter-helical salt bridges that stabilize the transporter in either one of the two conformations. In this model, after protons bind to carboxylate residues in the ExxER/K motif, in TMH7, or both, a large conformational change occurs resulting in the opening of the intracellular gate stabilized by a TMH4–TMH10 salt bridge that is disrupted. Once the protein is in the inward-open conformation, a second salt bridge between residues on TMH1 and TMH7 can form (Figure 2).

Here we analyze NPF sequences recovered from 42 sequenced plant genomes and examine the conservation of the four structural features implicated in the coordinated transport of protons and substrate in the NPF/POT families. Our data show that the ExxER/K motif is not strictly conserved in NPFs, with the percentage of NPFs with a completely conserved motif varying within each NPF subfamily as well as in different plant genomes. The glutamate on TMH7 is present only in two NPFs from a moss. Consequently, the potential inter-helical salt-bridge between TMH1 and TMH7 which involves the same glutamate residue, is only conserved in the two moss NPFs, but is absent in all other NPFs. The amino acids forming a second salt bridge between TMH4 and TMH10 are highly conserved in NPFs with some exceptions, in particular in one subfamily.

We combined these observations with results from functional studies that show that although NPFs mainly function as substrate/proton symporters, some NPFs have evolved to function as passive transporters or antiporters. This has led us to propose that not only have NPFs evolved different substrate specificities in plants, but may have also acquired alternate mechanisms for active or passive substrate transport that can be related to the presence or absence of specific structural features. We suggest that structural and functional studies are extended to NPFs lacking such features.

## Materials and Methods

### Sequence Acquisition From Fully Sequenced Plant Genomes

More than 100 plant genomes have been sequenced so far, but we limited our analysis to the genomes of the following 42 plants: *Amborella trichopoda, Ananas comosus, Aquilegia coerulea, Arabidopsis lyrata, Arabidopsis thaliana, Arachis duranensis, Brachypodium distachyon, Brassica rapa, Capsella rubella, Carica papaya, Cicer arietinum, Citrus clementina, Citrus sinensis, Cucumis sativus, Daucus carota, Erythranthe guttata, Eucalyptus grandis, Eutrema salsugineum, Fragaria vesca, Glycine max, Gossypium raimondii, Linum usitatissimum, Lotus japonicus, Manihot esculenta, Marchantia polymorpha, Medicago truncatula, Musa acuminata, Oryza sativa, Phaseolus vulgaris, Physcomitrella patens, Populus trichocarpa, Prunus persica, Ricinus communis, Selaginella moellendorffii, Setaria italica, Solanum lycopersicum, Solanum tuberosum, Sorghum bicolor, Theobroma cacao, Vitis vinifera, Zea mays*, and *Zostera marina*.

We also analyzed the genomes of the following green algae: *Bathycoccus prasinos, Chlamydomonas reinhardtii, Chlorella variabilis* NC64A, *Chromochloris zofingiensis, Coccomyxa subellipsoidea, Dunaliella salina, Micromonas pusilla* sp. RCC299, *Micromonas pusilla* sp. CCMP1545, *Ostreococcus lucimarinus, Ostreococcus tauri*, and *Volvox carteri*.

To recover the NPF sequences, we used the locus identification numbers reported in Léran et al. (2014) at Phytozome, the Plant Comparative Genomics portal of the Department of Energy’s Joint Genome Institute^1^. Retrieved sequences were confirmed using the NCBI BlastP suite^2^. We noticed that some of the sequences on the Phytozome database were truncated or had deletions, while sequences on NCBI seemed to be more complete. Some of the locus identification numbers listed in Léran et al. (2014) did not correspond to any entry, so the number of NPFs reported in this work may not correspond to that in Léran et al. (2014). We also used the sequences collected in von Wittgenstein et al. (2014). Homology searches were run using NPF sequences from different plants and subfamilies as queries both on Phytozome and on NCBI BlastP to find NPFs that may have not been identified previously. NPF sequences for the *Lotus japonicus* genome were obtained on http://www.kazusa.or.jp/lotus; since the *L. japonicus* genome is not present in the Phytozome or the NCBI databases, we were not able to double check the sequences obtained. Sequences for *A. duranensis* were obtained both on NBCI BlastP and the PeanutBase^3^. When an NPF member had not been named beforehand, we proceeded to assign it to a subfamily based on phylogenetic analysis and following the unified nomenclature. In particular, all NPFs from *A. trichopoda, A. comosus, A. duranensis, C. arietinum, D. carota, L. japonicus, E. guttata, M. acuminata, M. polymorpha, P. trichocarpa, R. communis*, and *Z. marina* were named here. Despite our best efforts to identify all NPF members in each genome, the quality and completeness of the sequencing data may have affected our analysis. For example, a new genome annotation of the *M. truncatula* genome (version Mt4.0) revealed several new NPFs that were not included in the previous Mt3.5 sequence release, bringing the total number of NPFs to 92, as reported in Pellizzaro et al. (2017). All sequences that were truncated at the N-terminus and/or lacking the ExxER/K motif were discarded and are not included in our analysis. In total, we collected 2383 NPF sequences (Supplementary Table 1). Based on sequence alignment and of the phylogenetic tree, some NPFs were reassigned to different subfamilies than in the Léran et al. (2014) paper. Newly named or renamed NPFs are marked with an asterisk (Supplementary Table 1).

### Multiple Sequence Alignment, Phylogenetic Trees, and Structural Modeling

Multiple sequence alignments of amino acid sequences were performed using the Clustal Omega program via the Web Services interface at the European Bioinformatics Institute (EMBL-EBI) (Sievers et al., 2011; McWilliam et al., 2013). We removed ambiguous sites from the alignment matrix using trimAl with the heuristic algorithm automated1 (Capella-Gutiérrez et al., 2009).

We used IQ-TREE (Trifinopoulos et al., 2016) as implemented on CIPRES (Miller et al., 2010) to find the best fit model of amino acid substitutions based on the Bayesian Information Criterion (Kalyaanamoorthy et al., 2017) and reconstructed the maximum-likelihood phylogenetic tree with 2000 Ultrafast Bootstrap replicates to access branch support (Hoang et al., 2018). The resulting phylogenetic tree was loaded into the Interactive Tree Of Life web-based tool for visualization^4^ (Letunic and Bork, 2016). The phylogenetic tree can be accessed at https://itol.embl.de/shared/FPS_2018.

Logos were created using WebLogo3^5^ (Crooks et al., 2004).

Protein structure homology modeling was performed using the SWISS-MODEL server^6^ (Arnold et al., 2006; Biasini et al., 2014).

## Results

### Distribution of the NPF Transporters in Plants

**FIGURE 3:**
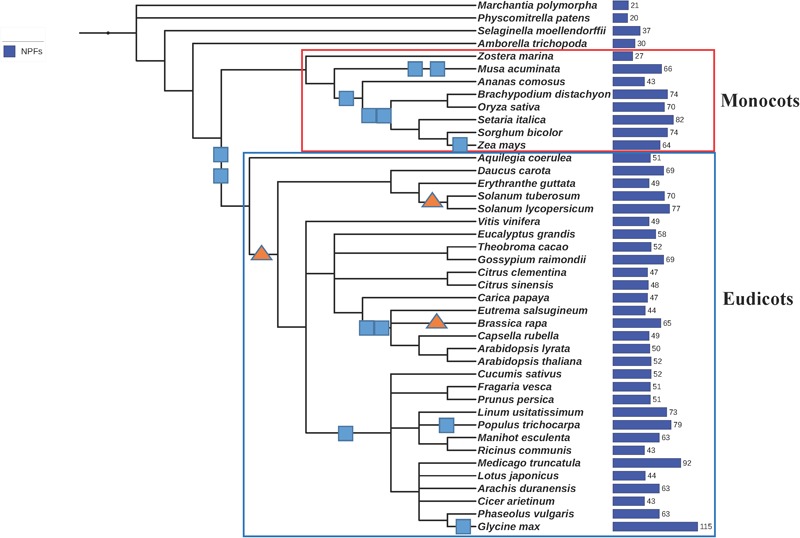
Phylogenetic tree of the 42 plants whose genomes were used in this study. We generated a phylogenetic tree from a list of taxonomic names of the 42 plants analyzed in this study using the online tool phyloT, a phylogenetic tree generator based on NCBI taxonomy (http://phylot.biobyte.de/). The tree was visualized using iTOL (http://itol.embl.de/). Whole genome duplications (WGD) are represented by *squares*, whole genome triplications are represented by *triangles* (based on Lee et al., 2012, 2017). The total number of NPFs contained in each genome is reported on the right. Monocots and eudicots are included in the *red* and *blue rectangles*, respectively.

We analyzed the genomes from 42 plants that were selected to provide a good coverage in the phylogeny of aquatic and land plants, monocots and eudicots (Figure 3). Included are the genome of the seagrass *Z. marina*, as well as genomes from ancient plants like the non-angiosperm land-plants *M. polymorpha*, a liverwort; *P. patens*, a bryophyte; and *S. moellendorffii*, a lycophyte. Also included is an ancestor of angiosperm plants, *A. trichopoda*. Genomes from closely related plants like *A. thaliana* and *lyrata* or *C. clementina* and *sinensis* were incorporated in the study to probe how NPFs have evolved more recently.

**FIGURE 4:**
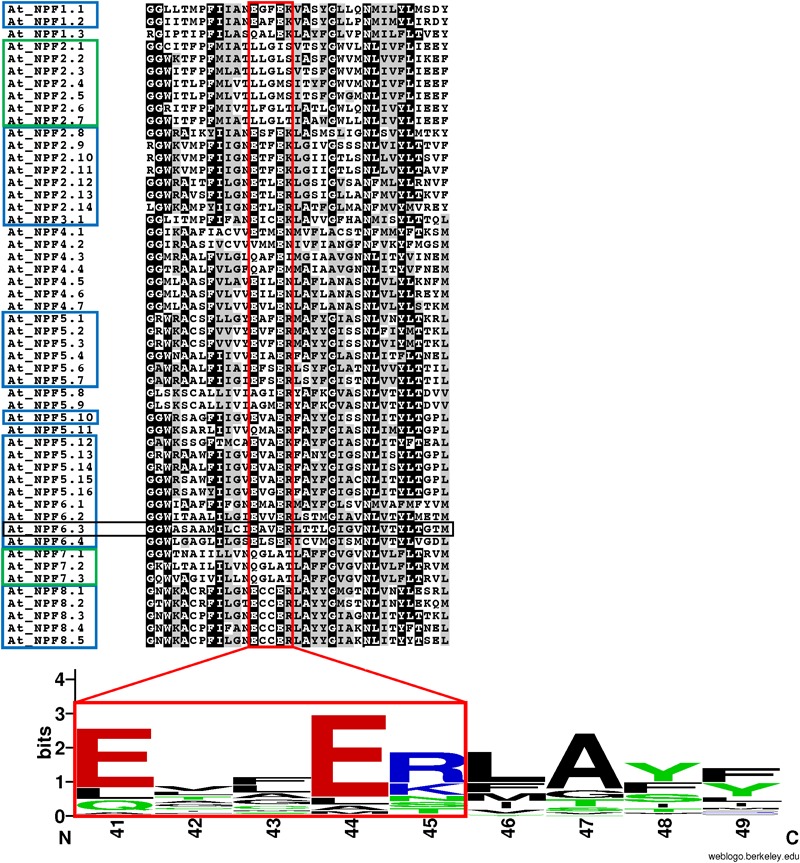
Conservation of the ExxER/K motif in *A. thaliana* NPFs. Multiple sequence alignment of the TMH1 region of 52 *Arabidopsis thaliana* NPFs. Included in the sequence are two glycine residues located N-terminal to the TMH1, but not part of the α-helix. The ExxER/K motif (*red box*) is fully conserved in 31 AtNPFs (60%) (*blue box*), while ten AtNPFs (19%) completely lack the motif (*green box*). A logo was created to represent the motif sequence conservation. Amino acid numbering is based on the sequence of AtNPF6.3. Amino acids are colored based on their chemical properties (hydrophobic amino acids are black, polar are green, basic are red, and acidic are blue). The overall height of stacks represents the sequence conservation while the height of letters indicates the relative frequency of each amino acid in that position (Crooks et al., 2004).

We collected and analyzed a total of 2383 NPF sequences (Supplementary Table 1). While most plants like *A. thaliana* have about 50 NPFs (Figure 4), the number of NPFs in the plant genomes ranges widely. Some genomes have as low as 20 and 21 NPFs like in the bryophyte *P. patens* and the liverwort *M. polymorpha*, respectively, to as high as 92 and 114 in the legumes *M. truncatula* and *G. max*, respectively (Supplementary Table 2). The high number of NPFs present in plants is in sharp contrast with the low number of the evolutionarily related POT/PTR/NRT1 transporters in other organisms. POTs comprise only one to four members in bacteria, one in yeast, and four in humans; algae have only one or two NRT1s; fungi have from one to seven PTRs. The high number of NPFs in plants can be related to the propensity of plants to expand their genomes by duplication or triplication events (Figure 3) (Lee et al., 2012, 2017). The high rate of retention of duplicated genes has been linked to benefits acquired during their long evolutionary history (Panchy et al., 2016). In the case of NPFs, new functions acquired during the long evolutionary history of plants including the expansion to a large number of different substrates may have resulted in high retention of the duplicated NPF genes (Corratgé-Faillie and Lacombe, 2017). Expanded roles and potential new mechanisms gained by NPFs have not been fully explored as functional studies have been limited to a small number of them.

**FIGURE 5:**
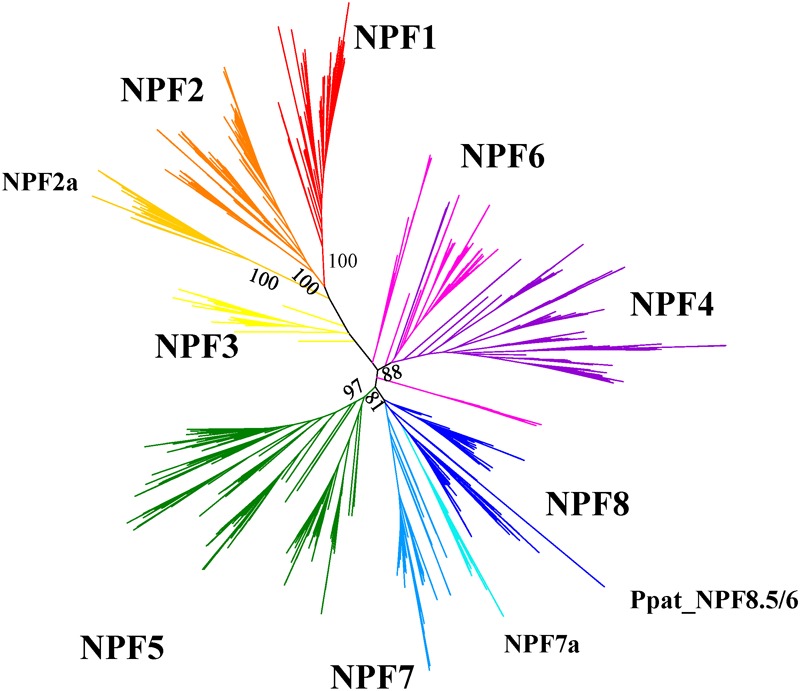
Unrooted maximum-likehood phylogenetic tree of 2383 NPFs sequences from 42 plants visualized with iTOL (Letunic and Bork, 2016). Eight subfamilies are represented with different colors and named based on the nomenclature proposed in Léran et al. (2014). Two subfamilies with specific characteristics regarding the ExxER/K motif, as shown in Figure 6, are labeled as NPF2a and NPF7a. Percent bootstrap values are given for the main branches and support the distribution in subfamilies. The phylogenetic tree can be accessed at https://itol.embl.de/shared/FPS_2018.

After we aligned our collection of sequences, we obtained a maximum likelihood phylogenetic tree that shows NPFs to be distributed in several clades supported by high bootstrap values (Figure 5; Supplementary Figure 1 can be expanded so that each NPF’s name is visible). Such clades correspond to the eight subfamilies previously identified by Léran et al. (2014) and to the 10 supergroups described by von Wittgenstein et al. (2014). In our analysis, we use the nomenclature proposed by Léran et al. (2014) as it has been widely accepted in subsequent publications.

We observed that NPFs are distributed unevenly in the subfamilies (Supplementary Table 2): some plants may completely lack NPFs in one subfamily, while others have a large number of NPFs in the same subfamily. One good example is represented by the NPF1 subfamily: genomes from *A. comosus, M. polymorpha, P. patens, S. moellendorffii*, and *Z. marina* lack NPF1 members, while *S. lycopernicum* and *S. tuberosum* have fifteen and nineteen NPF1s, respectively (Supplementary Table 2). *M. polymorpha, P. patens* and *S. moellendorffii* lack NPF transporters in the NPF2 subfamily, but twenty other plants have ten or more NPF2 members. In most genomes, the NPF3 subfamily contains the least members, for a total of 124 members in the 42 genomes analyzed. On the other end, the NPF5 subfamily has the largest number of members and a total of 567 members. Legumes *M. truncatula* and *G. max* have between one quarter and one third of their NPF members in the NPF5 subfamily: 29 of 92 for *M. truncatula* and 30 of 114 for *G. max.* However, this trend does not extend to legume *L. japonicus* which has only seven of its 44 NPFs in the NPF5 subfamily.

Our analysis led us to reassign some NPFs, as indicated in Supplementary Table 1 where NPFs moved to a different subfamily are marked with an asterisk. One example is represented by two NPFs from *P. patens* that in Léran et al. (2014) were assigned to the NPF6 family and named PpNPF6.1 and PpNPF6.2. Based on our alignment, instead, the two transporters belong to the NPF4 family. This is in agreement with the analysis by von Wittgenstein et al. (2014), as the same NPFs were located in the supergroup B along with PpNPF4.1. Therefore, we renamed PpNPF6.1 and PpNPF6.2 as PpNPF4.2 and PpNPF4.3, respectively.

### Survey of the ExxER/K Motif in NPFs

Many NPFs have been shown by functional studies to be proton-coupled transporters. Their ability to actively transport ligands across membranes requires amino acids with chargeable side chains that can bind protons. Residues that can fulfill this role have been identified in NPFs/POTs in the ExxER/K motif containing three chargeable amino acids on TMH1. The two glutamates and the arginine/lysine are highly conserved in bacteria and animal POTs, although some natural motif variants do occur where some chargeable amino acids are changed to neutral amino acids.

In our analysis, we used the AtNPF6.3’s sequence and its crystal structure as references. AtNPF6.3’s TMH1, where the ExxER/K motif is located, includes roughly 35 residues (WASAAMILCI**E_41_**AV**E_44_R_45_**LTTLGIGVNLVTYLTGTM) with the first glutamic acid in the motif, Glu41, occupying the 11th position of the TMH1. Therefore, the motif is located slightly closer to the cytoplasmic side of the membrane. The ExxER/K motif in AtNPF6.3 is conserved and when Glu41, Glu44 or Arg45 were mutated to an alanine, a loss of both nitrate uptake activity was observed in yeast- and oocyte-based nitrate uptake essays (Ho and Frommer, 2014; Sun et al., 2014).

The multiple sequence alignment of 52 *A. thaliana* NPFs shows that only 31 (60%) have a completely conserved ExxER/K motif (Figure 4). In contrast, ten (19%) completely lack chargeable amino acids in the corresponding region. These latter NPFs belong to two subfamilies, NPF2 and NPF7. Interestingly, the NPF2 subfamily contains both members with no chargeable amino acids (NPF2.1–2.7) and members that harbor a completely conserved ExxER/K motif (NPF2.8–2.14). Previously, AtNPF2.1–2.7 have been assigned to a subfamily called NAXT for NitrAte Excretion Transporter (Segonzac et al., 2007; Léran et al., 2014). All three AtNPF7 subfamily members lack chargeable amino acids.

After aligning 2383 NPF sequences from 42 plants, we identified the ExxER/K motif in each transporter using the AtNPF6.3 sequence as a reference. To help correctly identify the TMH1 in multiple sequence alignment, we included two conserved glycine residues in positions -1 and -2 with respect to the first amino acid of the AtNPF6.3’s TMH1. Both glycine residues located N-terminal to the TMH1 are conserved in 1455 NPF sequences and at least one of the two glycine residues is conserved in all but 72 NPFs, allowing us to unambiguously identify the residues corresponding to TMH1 and the ExxER/K motif (Supplementary Table 1, column D and Supplementary Figure 2. Supplementary Figure 2 contains the ExxER/K motif and percent bootstrap values obtained for the phylogenetic tree). Of the 2383 NPFs sequences analyzed, 1469 (62%) contain a completely conserved ExxER/K motif (Table 2 and Supplementary Table 2). It is worth noting that some NPFs, mostly belonging to the NPF3 subfamily, contain the anomalous Exx**D**R/K motif and a few have a **D**xx**D**R/K motif: these are counted as conserved. In contrast, 326 (14%) NPFs completely lack any chargeable amino acids in the TMH1 region that aligns with the motif.

**Table 2:** Summary of the distribution of NPFs in subfamilies and conservation of structural features.

NPF SubFamily	Total NPFs	Conserved ExxER/K (%)	Conserved TMH4–TMH10 salt bridge (%)
**NPF1**	204	147 (72%)	156 (76%)
**NPF2**	258	248 (96%)	237 (92%)
**NPF2a**	94	0 (0%)	84 (89%)
**NPF3**	124	124 (100%)	114 (92%)
**NPF4**	367	0 (0%)	358 (97%)
**NPF5**	567	464 (82%)	482 (85%)
**NPF6**	265	265 (100%)	257 (97%)
**NPF7**	181	0 (0%)	0 (0%)
**NPF7a**	41	0 (0%)	39 (95%)
**NPF8**	282	221 (78%)	244 (86%)
**Total**	2383	1469 (62%)	1974 (83%)

*Phylogenetic analysis was used to assign each of the 2383 NPFs from our collection to one of eight subfamilies proposed in Léran et al. (2014) or to one of two new subfamilies proposed in this paper. Each subfamily contains a different number of members (column 2). The conservation of two structural features, the ExxER/K motif (column 3) and the TMH4–TMH10 salt bridge (column 4), are listed for each subfamily. In the NPF6 subfamily all members have a completely conserved ExxER/K motif, while the NPF4 and NPF7 subfamilies lack members harboring the motif. More than half of the NPF3 members contain the anomalous E/DxxDR/K motif which we counted as conserved. A high percentage of NPFs harbor residues that can potentially form the TMH4–TMH10 salt bridge, with the exception of all members of the NPF7 subfamily.*

Although these numbers are very similar to what we observed in *A. thaliana*, the percentage of NPFs having a perfectly conserved ExxER/K motif is not the same in different genomes. For instance, in *S. italica*, only 44% of NPF proteins have a completely conserved motif; similarly, in *S. bicolor* and *O*. *sativa* only about 49% NPFs retain the motif completely. Some plants have a much higher percentage of completely conserved motif: for example, 75% in *B. rapa* and 73% in *A. trichopoda* and *M. esculenta* (Supplementary Table 2).

**FIGURE 6:**
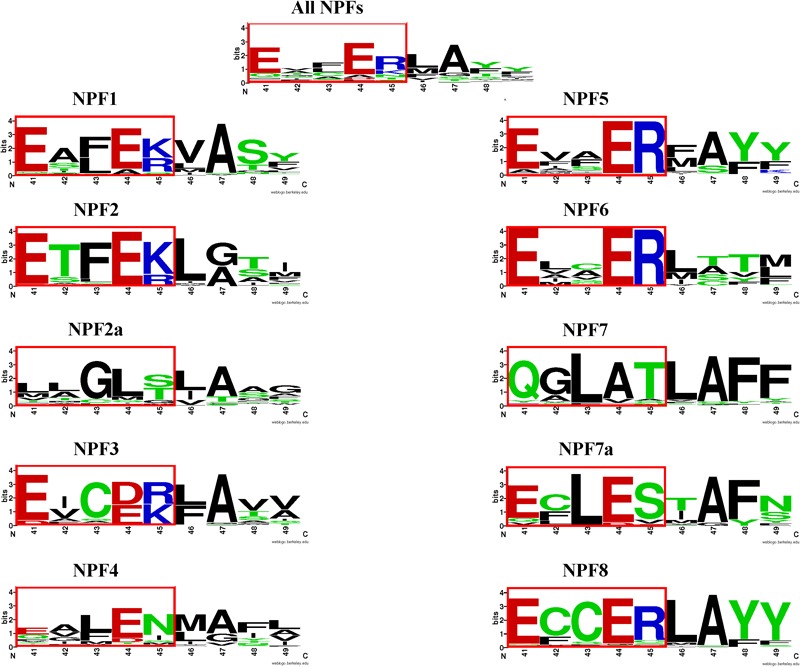
LOGOS of the ExxER/K motif sequence in the NPF subfamilies. Graphical representation of sequence conservation with the transmembrane helix 1 (TMH1) using all NPF sequences or NPF sequences from each subfamily separately. Numbering and coloring as in Figure 4.

When we examined the distribution of the ExxER/K motif in the eight NPF subfamilies, we observed that it differs with subfamilies, as summarized in Table 2. At one extreme, 100% of NPF6 and NPF3 subfamily members harbor a conserved ExxER/K motif. At the other extreme, all NPF4 and NPF7 members have lost at least one of the chargeable amino acids in the ExxER/K motif with 79 of 368 (21%) of the NPF4 members and 179 of 222 (80%) of the NPF7 members lacking all the chargeable amino acids. NPF members from the other subfamilies fall between the two extremes represented by the NPF6/NPF3 and the NPF4/NPF7 subfamilies (Table 2 and Supplementary Table 2). Sequence conservation of the ExxER/K motif region in each subfamily was graphically visualized through logos (Figure 6). The logos clearly show that each subfamily has evolved a characteristic motif sequence.

Interestingly, although all NPF3 subfamily members have a completely conserved ExxER/K motif, they harbor some variations (Supplementary Table 1). About half of the NPF3s have an aspartic acid instead of a glutamic acid in the fourth position and show a preference for an arginine in the last position, resulting in the ExxDR motif, with a few NPF3s from monocots harboring the rare DxxDR motif. The other half have the more common glutamic acid in the fourth position with a preference for a lysine in the fifth position and therefore an ExxEK motif. In the phylogenetic tree the NPF3 members harboring the E/DxxDR motif are separated from the ones with the ExxEK motif, indicating a different evolutionary history (Supplementary Figure 2).

We observed that NPFs that have been assigned to the NPF2 subfamily contain members that harbor a conserved motif as well as members that lack chargeable amino acids in the ExxER/K motif. Analysis of the phylogenetic tree revealed that the NPFs with different ExxER/K motifs are located in separated branches with high percent bootstrap values (Figure 5 and Supplementary Figure 2). Similarly, the NPF7 subfamily have members that completely lack any chargeable amino acids, while other NPFs harbor an ExxER/K motif that contains either one or both glutamates. These NPF7s are located in different branches of the phylogenetic tree (Figure 5 and Supplementary Figure 2). We discuss these two families in more detail below.

### The ExxER/K Motif in NPF2 Members

The *A. thaliana* genome contains 14 NPF2s, seven of which, AtNPF2.1–2.7, are organized in a cluster on chromosome 3. These latter NPFs share a high level of sequence similarity, suggesting they are derived from recent duplication events. AtNPF2.7 was the first to be characterized and was named NAXT1 for NitrAte Excretion Transporter based on its ability to mediate nitrate efflux in roots (Segonzac et al., 2007). AtNPF2.7 was shown to be a passive nitrate transporter in oocytes (Segonzac et al., 2007). Because of their high sequence conservation, the remaining NPF2 members in the cluster have been referred to as NAXT proteins as well. Recently some of them have been functionally characterized showing that AtNPF2.3 is a passive nitrate transporter like AtNPF2.7 (Taochy et al., 2015), while AtNPF2.4 and AtNPF2.5 passively transport chloride with no indication that either can also transport nitrate (Li et al., 2016; Li B. et al., 2017). In a phylogenetic tree, AtNPF2.1–2.7 form a distinct subfamily (Segonzac et al., 2007; Léran et al., 2014) that we propose to rename NPF2a (Figure 5). Analysis of the seven AtNPF2a members shows that they all lack protonatable amino acids in the ExxER/K motif (Figure 4). This is in agreement with functional studies in which at least four of the NPF2a members in *A. thaliana* have been shown to be nitrate/chloride passive transporters (Segonzac et al., 2007; Taochy et al., 2015).

The other seven AtNPF2 members, AtNPF2.8-2.14, do not belong to the NAXT/NPF2a family, and are grouped in a distinctly separated clade in the phylogenetic tree (Figure 5). These NPF2s have a perfectly conserved ExxER/K motif and are therefore expected to be proton-dependent transporters (Figure 4). Functional studies performed with AtNPF2.13, AtNP2.12, and AtNPF2.9 showed that they are indeed proton-dependent nitrate transporters (Almagro et al., 2008; Fan et al., 2009; Wang and Tsay, 2011), while AtNPF2.10 and AtNPF2.11 are proton-coupled glucosinolate symporters (Nour-Eldin et al., 2012; Jørgensen et al., 2015).

Analysis of the phylogenetic tree for our collection of NPFs shows that the 352 NPF2 members from 39 sequenced plant genomes (*M. polymorpha, P. patens*, and *S. moellendorffii* lack NPF2 proteins) are distributed into two distinct subfamilies, similar to what was observed for the NPF2 members from *A. thaliana*. The NPF2a subfamily contains a total of 94 transporters (including AtNPF2.1–2.7) (light orange branches in Figure 5). All NPF2a members, excepting one from *D. carota* which has a glutamate in the first position of the motif, completely lack chargeable amino acids in the motif (Supplementary Table 3). The logo created for the 94 NPF2a proteins using the sequences corresponding to the **E**xx**ER**/**K** motif shows that the motif is changed to **L**LG**LS/T** (Figure 6). The genome of *A. trichopoda*, an ancient angiosperm that stands alone as a monophyletic sister to the existent angiosperms, lacks members in the NPF2a subfamily. Among monocots, *Z. mays* and *Z. marina* lack NPF2a members; *S. bicolor, A. comosus, O. sativa*, and *B. distachyon* have one NPF2a protein each; *M. acuminata* have two and *S. italica* has four, one on chromosome 5 and three on chromosome 3. With the exception of *L. usitatissimum, P. vulgaris, C. clementina*, and *C. sinensis*, all eudicot genomes contain at least one member in the NPF2a subfamily. In *A. lyrata*, from which the self-fertilizing species *A. thaliana* diverged about 10 million years ago, the five NPF2a proteins are clustered on chromosome 5 which corresponds to chromosome 3 in *A. thaliana* (Hu et al., 2011). This suggests that NPF2a members had already duplicated before speciation of *A. thaliana* and *A. lyrata*. NPF2a members are clustered on the same chromosome in other genomes, like *M. truncatula* and *S. tuberosum*. *S. lycopersicum*, instead, has eight NPF2a members clustered into two different chromosomes, three on chromosome 11 and five on chromosome 6. From these observations, it appears that NPF2a proteins may have evolved later in plant evolution but predating monocotyledon-dicotyledon divergence.

Most NPF2 members including AtNPF2.8-2.14 (for a total of 258 NPF2s) are located in a larger clade (dark orange branches in Figure 5). Their TMH1 sequences contain a completely conserved ExxER/K motif (Supplementary Figure 2) as graphically evident in their logo (Figure 6). An exception is represented by a group of five NPF2s, all from grasses, that although completely lacking chargeable amino acids in the ExxER/K motif - their motif is changed to **Y**/**F**AA**AS** - do not belong to the NPF2a subfamily (yellow branches in Supplementary Figure 2). Their separation from the NPF2a subfamily suggests a different evolutionary origin.

### The ExxER/K Motif in NPF7 Members

In *A. thaliana*, the three NPF7s are located in three different chromosomes and they are phylogenetically distant from each other as evident from their position in the phylogenetic tree (Supplementary Figure 2, red labels). In the three AtNPF7s the **E**xx**ER**/**K** motif is changed to **Q**GL**AT** (Figure 4). Functional studies in oocytes indicated that both AtNPF7.3 and AtNPF7.2 are low-affinity proton-coupled transporters (Lin et al., 2008; Li et al., 2010). AtNPF7.3 and AtNPF7.2 are both nitrate xylem transporters that have been shown to control root-to-xylem nitrate loading and unloading, respectively, and work together to fine-tune nitrate transport from roots to shoots (Lin et al., 2008; Li et al., 2010; Chen et al., 2012). Additionally, AtNPF7.3 can transport nitrate in both directions (Lin et al., 2008). The observation that AtNPF7.3 and AtNPF7.2 are proton-dependent nitrate transporters is surprising as they do not have chargeable amino acids in the region corresponding to the ExxER/K motif that would carry out proton-binding necessary for proton transport. However, a recent study carried out in oocytes using AtNPF7.1, AtNPF7.2, and AtNPF7.3 as well as their orthologs from rice, OsNPF7.9, and from maize, ZmNPF7.10 is challenging the previous results as the authors were not able to show a strong transport activity for nitrate in oocytes. Instead, they demonstrated that these NPFs function in K^+^ efflux with simultaneous influx transport of protons, effectively working as K^+^/H^+^ antiporters (Li H. et al., 2017).

When we analyzed the sequences assigned to the NPF7 subfamily by Léran et al. (2014), we observed that all 42 plant genomes contain at least one NPF7 member that lacks chargeable amino-acids in the ExxER/K motif, for a total of 178 NPF7s (Supplementary Figure 2). The remaining 44 NPF7s do contain one or two glutamic acids, but none of them have an arginine or lysine in the fifth position of the motif. In our phylogenetic tree almost all NPF7s without chargeable amino acids belong to a different clade from the ones with at least one chargeable amino acid (Figure 5). This observation is in agreement with the von Wittgenstein phylogenetic analysis, where these NPF7s are assigned to supergroup F, while the other NPF7s belong to supergroup G (von Wittgenstein et al., 2014) (Figure 5). We propose to name the subfamily NPF7a.

The different phylogenetic origin of the two NPF7 groups correlates with their ExxER/K motif conservation. The logo obtained using the region corresponding to the motif for NPF7 subfamily members shows a new motif, **Q**GL**AT**, as in the three *A. thaliana* NPF7s (Figure 6). Interestingly, a nine amino acid sequence, **Q**GL**AT**LAFF, is completely conserved in 106 NPF7 members. This suggests a common origin and an evolutionary need to maintain such a motif. In contrast, the ExxER/K region logo for the NPF7a subfamily members shows that the motif has become **E**CL**ES** (Figure 6). Interestingly, all the NPF7a members belong to seven plants, five of which are grasses (*B. distachyon, O. sativa, S. italica, S. bicolor* and *Z. mays*), and two (*M. acuminata* and *A. comosus*) belong to the Bromelliaceae family, that diverged from the grass family 100 million years ago. The *O. sativa* genome contains ten NPF7s, seven of which, belong to the NPF7a subfamily. Some OsNPF7a members have been studied. OsNPF7.2 was functionally characterized in oocytes and shown to be a low affinity nitrate transporter that is not pH dependent (Hu et al., 2016; Wang et al., 2018). OsNPF7.3 was shown to be a di/tripeptide transporter and rice growth is enhanced when OsNPF7.3 is highly expressed (Ouyang et al., 2010; Fan et al., 2014; Fang et al., 2017): no functional studies have been performed with this transporter. Finally, two splicing variants of OsNPF7.7 were shown to regulate shoot branching and nitrogen utilization efficiency (Huang et al., 2018): overexpression of OsNPF7.7-1 could promote nitrate influx and concentration in root, whereas overexpression of OsNPF7.7-2 could improve ammonium influx and concentration in root. Additional functional studies are needed to clarify if NPF7a subfamily members can transport protons.

### Survey for the TMH7 Proton-Binding Site

In addition to the glutamate residues located on the ExxER/K motif that are considered to be the principal residues responsible for proton binding and transport, some bacterial POTs contain an additional amino acid associated with proton transport, a glutamate residue located on TMH7 (Figure 2). Although this residue is not strictly conserved in bacterial POTs, when it was mutated in PepT1_St_, proton driven uptake was affected (Solcan et al., 2012). Sequence and structural alignments show that the corresponding residue in AtNPF6.3 is an alanine, Ala357 (Figure 1C). When we surveyed our collection of NPF sequences, we found that this structural feature is missing from all plant NPFs with the exception of NPF8.5 and NPF8.6 from *P. patens* which harbor an aspartate at the position corresponding to the bacterial TMH7 glutamate.

### Residues Involved in Inter-helical Salt Bridges

The role of salt bridges in orchestrating structural changes during substrate transport has been discussed for several POT proteins (Terada and Inui, 2012; Yan, 2013, 2015; Newstead, 2015, 2017). In particular, two salt bridges have been implicated in the alternating-access cycle and form either in the outward- or the inward-open conformations: the TMH4–TMH10 and the TMH1–TMH7 salt bridges, respectively (Figure 2).

The TMH1–TMH7 salt bridge has been observed in some bacterial POTs that were crystallized in the inward-open conformation (Newstead et al., 2011; Solcan et al., 2012; Boggavarapu et al., 2015; Parker et al., 2017). The AtNPF6.3 crystal structure was obtained in the same conformation, but the salt bridge is absent as the residues that form it are a glycine (Gly52) and an alanine (Ala357) (Figure 1C). We mutated these amino acids *in silico* to an arginine and a glutamate, respectively, and we observed that their side chains are within hydrogen bonding distance and may therefore form a salt bridge, confirming the correctness of the alignment (Figure 1D).

In contrast, residues that form the TMH4–TMH10 salt bridge in the outward-open conformation are conserved in AtNPF6.3 and the salt bridge can potentially form between Lys164 on TMH4 and Glu476 on TMH10, as proposed (Parker and Newstead, 2014). Since AtNPF6.3 as well as all the bacterial POTs were captured in the inward-open conformation in the crystal structures, the TMH4–TMH10 salt bridge has not been directly observed, but only predicted to form in the outward-open conformation (Parker and Newstead, 2014; Sun et al., 2014).

When we examined the level of conservation of the residues that form the two salt bridges in our collection of NPF sequences, we found that the oppositely charged amino acids required to form the TMH1–TMH7 salt-bridge are not present in any NPF, with the only exception represented by moss PpatNPF8.5 and PpatNPF8.6. Both their sequences contain a lysine in TMH1 and an aspartate in TMH7 that align with the residues that form the TMH1-TMH7 salt bridge in the inward-facing crystal structures of *S. oneidensis* PepT1, *S. thermophiles* PepT1, *Y. enterocolica* PEPT, and *X. campestris* PepT1 (Newstead et al., 2011; Solcan et al., 2012; Boggavarapu et al., 2015; Parker et al., 2017). The fact that only two NPFs from a moss retain the residues required to form this salt bridge suggests that these residues were lost very early in the evolution of the NPFs.

Our analysis of the residues that can form the salt-bridge between TMH4 and TMH10 reveals that they are more conserved than the ones required for the TMH1-TMH7 salt-bridge. Of 2383 NPF sequences, 1275 NPFs have a lysine and 791 NPFs an arginine in the position corresponding to Lys164 in AtNPF6.3, totaling 2066 positively charged amino-acids or 87% conserved positive residues. And, 1401 sequences have a glutamic acid and 859 an aspartic acid in the position corresponding to Glu476 in AtNPF6.3, for a total of 2260 conserved negative residues or 95% (Supplementary Table 1). Therefore, the negatively charged amino acid on TMH10 is more conserved than the positively charged one on TMH4. A total of 1971 (83%) of the NPF sequences analyzed harbor two oppositely charged amino acids that can potentially form the TMH4–TMH10 salt bridge (Table 2). When we looked at the distribution of arginine and lysine residues in different subfamilies where two oppositely charged amino acids are present, we observed that while most NPF1, NPF2 and NPF3 subfamily members have an arginine, NPF4, NPF6 and NPF8 members mostly have a lysine residue. This has potential importance to structure and molecular mechanism as the geometry of salt bridges is different for arginine and lysine residues (Donald et al., 2011).

The presence of two oppositely charged residues on TMH4 and TMH10 varies in the eight subfamilies identified by Léran et al. (2014) and the two new NPF2a and NPF7a subfamilies, as summarized in Table 2. The NPF subfamilies with most members able to form the TMH4–TMH10 salt bridge are NPF6 and NPF4 with 97% of proteins harboring the two oppositely charged amino-acids, followed by NPF3 with 92%. Among subfamilies, the NPF7 one presents a peculiar situation. Strikingly, out of 222 members of the NPF7 subfamily, only 39 harbor oppositely charged amino-acids in the positions required for the TMH4–TMH10 salt bridge formation (Table 2). These latter NPF7s belong to the NPF7a subfamily that we defined above and therefore also contain at least one or two glutamic acids in the ExxER/K motif (Figure 5, light blue branches; Supplementary Figure 2). The remaining NPF7s lack one or both the amino acids that form the salt bridge between TMH4 and TMH10 and some even have two amino acids of the same charge. As observed before, the NPF7 subfamily members that are separated from the NPF7a subfamily also lack chargeable amino acids in the ExxER/K motif (Figure 5, dark blue branches; Supplementary Figure 2). It is intriguing that these NPFs lack amino acids implicated in the TMH4–TMH10 salt bridge and as well as chargeable amino-acids in the ExxER/K motif, thus missing both structural features that are at the basis of the proposed alternating-access mechanism for NPF transporters.

### Genomic Survey of Nitrate Transporters in Algae

Green algae (phylum Chlorophyta) are a highly diverse group of photosynthetic eukaryotes from which the terrestrial plant lineage emerged > 1 billion years ago and therefore are phylogenetically closely related to plants (Heckman et al., 2001). The algal proteins related to NPFs/POTs are called NRT1 and transport nitrate/nitrite (Sanz-Luque et al., 2015).

As previously reported, sequenced genomes from Chlorophytes contain one or two NRT1s, or none at all (Sanz-Luque et al., 2015). *Chromochloris zofingiensis* contains two NRT1s, while *Chlorella variabilis* NC64A and *Coccomyxa subellipsoidea* both contain one full and one partial NRT1; *Bathycoccus prasinos, Chlamydomonas reinhardtii, Dunaliella salina, Ostreococcus lucimarinus, Ostreococcus tauri*, and *Volvox carteri* have a unique NRT1; *Micromonas pusilla sp. RCC299* contains one NRT1, while *Micromonas pusilla sp. CCMP1545* does not contain any NRT1. None of the algal NRT1s has been functionally characterized so far.

Chargeable amino acids in the ExxER/K motif as well as the oppositely charged amino acids required to form the TMH4–TMH10 salt bridge are conserved in all algal NRT1s (Supplementary Table 1). In contrast, the glutamate on TMH7 is only found in NRT1s from *B. prasinos, M. pusilla, O. lucimarinus* and *O. tauris*, which are all Mamiellophyceae, a class of green algae. These latter NRT1s also have an arginine on TMH1 that can form the TMH1-TMH7 salt bridge with the TMH7 glutamate. When aligned with our collection of plant NPFs sequences, the Mamiellophyceae NRT1s form a distinct subgroup with two NPFs from the moss *P. patens*, PpatNPF8.5 and PpatNPF8.6 (Supplementary Figure 3) which are the only plant NPFs to harbor the TMH1-TMH7 salt bridge residues.

## Discussion

NPF transporters are part of the major facilitator superfamily of secondary active transporters and are phylogenetically related to POT, PTR, NRT1 transporters of bacteria, animals and algae (Newstead, 2015, 2017; Drew and Boudker, 2016). Among these transporters, plant NPF transporters are recognized as uniquely able to transport a wide range of substrates across membranes as proton-coupled symporters (Léran et al., 2014; Corratgé-Faillie and Lacombe, 2017). The crystal structure of one NPF transporter, AtNPF6.3 was solved in 2014 (Parker and Newstead, 2014; Sun et al., 2014). It shows conservation of structure with the phylogenetically related POT transporters of bacteria for which crystal structures are also available. These transporters are thought to use an alternating access mechanism, similar to other secondary active transporters (Newstead, 2015, 2017; Drew and Boudker, 2016). In this mechanism, transport is initiated when the protein is in the outward-open conformation by protons binding to chargeable amino acids belonging to the ExxER/K motif on TMH1 and a D/E residue on TMH7. This conformation is stabilized by a salt bridge formed by oppositely charged residues on TMH4 and TMH10, which also serves as an intracellular gate. Simultaneous or subsequent substrate binding causes conformational changes that lead to the formation of the occluded state. Proton movement toward the salt bridge then facilitates the opening of the intracellular gate, forming the inward-open conformation and the release of substrate and protons to the cytoplasm. In some POTs, the inward-open conformation is stabilized by a salt bridge formed between the TMH7 D/E residue and an oppositely charged K/R residue on TMH1. Subsequently, the protein resets to the outward-open conformation, ready to start another transport cycle (Figure 2) (Newstead, 2015, 2017; Drew and Boudker, 2016).

In this study, we analyzed 2383 NPF sequences from 42 plant genomes including those from monocot and eudicot land plants as well as aquatic plants. The NPFs were surveyed for sequence conservation of four structural features implicated in the proposed proton-driven transport mechanism: the chargeable amino acids in the ExxER/K motif on TMH1, the D/E residue on TMH7, and the oppositely charged amino acids responsible for forming the inter-helical salt bridges between TMH1 and TMH7 and between TMH4 and TMH10.

### The ExxER/K Motif and Proton-Binding Residues in TMH1 in NPFs/POTs

Our analysis shows that the chargeable amino acids of the ExxER/K motif are conserved in only 62% of plant NPFs (Table 2). The level of conservation differs in different NPF subfamilies with some subfamily members completely lacking chargeable amino acids in the region corresponding to the motif. We observed that members of some subfamily have evolved distinct signature motifs. In particular, two subfamilies, NPF2 and NPF7 have a peculiar distribution of the ExxER/K motif within their members. Most members of the NPF2 subfamily contain a conserved ExxER/K motif, but some NPF2 members completely lack chargeable amino acids in the corresponding region, and are thus unable to bind protons necessary for the proposed mechanism. These latter NPF2s form a subfamily that we propose to name NPF2a. The NPF2a subfamily contains 94 members (Supplementary Table 2): some plants have only one member in the NPF2a subfamily, but others have several members. In genomes for which data is available, the genes for NPF2a members are clustered on the same chromosome, indicating recent duplication events. Several *A. thaliana* members of the NPF2a subfamily have been functionally characterized and shown to be passive nitrate or chloride transporters implicated in root-to-shoot efflux (Segonzac et al., 2007; Taochy et al., 2015). We speculate that NPF2a subfamily members we identified in other plants may also be passive transporters. We hypothesize that NPF2a members evolved into passive transporters from an ancestor lacking protonatable amino acids in the ExxER/K motif. Without chargeable amino acids, the ancestor lost its ability to bind and transport protons necessary to fuel the alternating-access mechanism and could only function as a passive transporter. Subsequent duplications resulted in several transporters per genome that then evolved to passively transport different substrates. Additional functional studies are needed to support this hypothesis.

Another interesting case is represented by the NPF7 subfamily. When we examined the sequences in this subfamily, we observed that most NPF7s completely lack chargeable amino acids in the ExxER/K motif. There is controversy over the biochemical activities displayed by the several *A. thaliana, O. sativa*, and *Z. mays* NPF7 transporters in this group that have been studied, with observations of proton-driven nitrate symport as well as proton-potassium antiport (Lin et al., 2008; Li et al., 2010, Li H. et al., 2017; Chen et al., 2012). Our data leads us to predict that with the ExxER/K motif missing in these transporters, proton-driven symport would not occur, or if it occurs, there must be another mechanism for binding protons. We also observed that some NPF7 members contain one or two glutamates of the motif, resulting in the ExxES consensus sequence. These NPF7s are exclusively from monocots and in the NPF phylogenetic tree are grouped in a subfamily that we named NPF7a. Functional studies of an NPF7a transporter from rice have shown it to be a low affinity nitrate transporter, with nitrate uptake not dependent on proton transport. Further functional studies are needed to clarify the role of NPF7a proteins in monocots.

Overall, our results suggest there exists a correlation between the presence of an ExxER/K motif and secondary active transport; i.e., transport of a substrate driven by the concomitant transport of protons down their energetic gradient. NPF transporters lacking the ExxER/K motif have lost one of the key elements of the proposed mechanism for active transport and thus function as passive transporters.

The glutamate/aspartate residue located on TMH7 is available as a proton-binding site in the outward-open conformation in some bacterial POTs. Although this residue is not strictly conserved in bacterial POTs, when it was mutated in PepT1_St_, proton driven uptake was affected (Solcan et al., 2012). The corresponding residue in AtNPF6.3 is an alanine, Ala357 (Figure 2C). Analysis of the NPF sequences shows that this residue is not conserved in plant NPFs. The only exception is represented by two NPFs from a moss that do harbor an aspartate in a conserved position on TMH7. Thus, our data suggest that plant NPFs requiring protons for substrate transport mainly use the residues located on the ExxER/K motif.

### Residues That Can Form Salt Bridges in NPFs/POTs

Two sets of inter-helical salt bridges have been described in POTs that are important for the alternating-access mechanism. One salt bridge has been observed in the inward-open conformation between residues located on TMH1 and TMH7; a second salt bridge is predicted to form in the outward-open conformation between THM4 and THM10 (Figure 2) (Newstead, 2017). Amino acids forming the first salt bridge are not very conserved, while those forming the second one are highly conserved in bacterial and animal POTs. Our data shows that the two salt bridges are differently conserved in NPF transporters as well. The residues underpinning the TMH1–TMH7 salt bridge are absent in all plant NPFs with the exception of PpatNPF8.5 and PpatNPF8.6 where an aspartate residue on TMH7 can form a salt bridge with an arginine on TMH1. These latter NPFs align with some algal NRT1s that also have residues forming the salt bridge and therefore may represent a more ancestral form. In contrast, the THM4–THM10 salt bridge is conserved in most NPF subfamilies, with the curious exception of the NPF7 subfamily with only 17% of its members having the appropriate residues that form the salt bridge. These NPF7 members belong to the NPF7a subfamily that also contains at least one glutamate residue in its ExxER/K motif, as discussed above.

The difference in level of conservation of the residues involved in the formation of the two salt bridges is in agreement with the observation that the largest conformational change is from outward-open to occluded, rather than from occluded to inward-open (Newstead et al., 2011; Doki et al., 2013; Zhang et al., 2015; Lee et al., 2016). Thus, the TMH4–TMH10 salt bridge may be essential to the alternating access mechanism, while the TMH1–TMH7 salt bridge may only be necessary in a few POT/PRT/NRT1/NPF transporters. Interestingly, while the TMH1–TMH7 salt bridge has been observed in the crystal structures of POTs from *S. oneidensis, S. thermophilus, Y. enterocolica*, and *X. campestris* (Newstead et al., 2011; Solcan et al., 2012; Boggavarapu et al., 2015; Parker et al., 2017), the TMH4–TMH10 salt bridge has never been observed as there are no available crystal structures solved in the outward-open conformation.

### Algal NRT1 Transporters

We examined the four structural features implicated in NPF/POT/PRT proton-driven substrate transport in algal NRT1 transporters and found that the TMH1 ExxER/K motif and the residues contributing to the TMH4–TMH10 salt bridge are strictly conserved in all algal NRT1s. In contrast, the glutamate on TMH1 and the residues contributing to the TMH1–TMH7 salt bridge are only conserved in NRT1s from Mamiellophyceae algae. These NRT1s align with two NPFs from *P. patens* which are the only plant NPFs to harbor the residues forming the TMH1-TMH7 salt bridge (Supplementary Figure 3).

None of the algal NRT1s has been functionally characterized so far, but based on the conservation of two essential structural features, we speculate that, most likely, the NRT1s actively transport substrate in a proton-driven symport mechanism. This is significant because it suggests that these features are ancient and that during algal evolution the mechanism of proton-driven transport has been preserved.

### NPFs Are Versatile Transporters That Evolved New Functions

NPFs, like POTs, PTRs, and NRT1s, belong to the MFS superfamily that comprises facilitators, symporters, and antiporters, which move substrates across membranes via facilitated diffusion, cotransport, or exchange, respectively (Yan, 2013). POTs have been found to be proton-dependent transporters. Their crystal structures together with that of AtNPF6.3 have been used with genetic and biochemical studies to develop the alternating access model and to identify potential critical residues in NPFs/POTs/PTRs/NRT1s for mechanism. Mutagenesis studies have shown that when chargeable amino acids of the ExxER/K motif in several POTs and NPFs were mutated individually, the electrogenic transport of substrate was disrupted resulting in an inactive protein (Solcan et al., 2012; Doki et al., 2013; Ho and Frommer, 2014; Parker and Newstead, 2014; Sun et al., 2014; Jørgensen et al., 2015). Based on these experiments, these residues seem to play an irreplaceable role for proton-driven substrate symport. However, our results show that many NPFs lack some or even all the amino acids implicated in proton-driven active transport. Still, these proteins play essential roles in their respective plant’s life. Indeed, NPFs that lack chargeable amino acids in the ExxER/K motif have been shown to function as efflux transporters of either nitrate or chloride without simultaneous transport of protons (Segonzac et al., 2007; Tsay et al., 2007; Taochy et al., 2015; Léran et al., 2013; Li et al., 2016; Li B. et al., 2017). In this context, it is notable that the proton-driven dual-affinity influx nitrate transporter AtNPF6.3 has been shown to be able to mediate nitrate efflux in the 5.5–7.5 pH range with no proton transport involved (Léran et al., 2013). Thus, it would appear that at least some NPFs can constitutively function as passive substrate transporters or channels, even in the presence of a perfectly conserved proton-binding ExxER/K motif. Mutagenesis studies in NPFs and POTs have shown that, when the glutamate residues in the ExxER/K motif are mutated to neutral residues, counter-flow transport, that is not dependent on proton transport, can be still observed (Solcan et al., 2012; Doki et al., 2013; Sun et al., 2014; Zhao et al., 2014; Jørgensen et al., 2015). Based on these observations, we speculate that the capacity to function as a bidirectional transporter may be an intrinsic feature of NPFs. This feature may be beneficial to plants in certain ionic conditions. We propose that some transporters have evolved to be exclusively passive transporters, potentially through the loss of the ExxER/K motif. It has not been determined if the lack of chargeable amino acids makes passive efflux more effective. However, there are examples of NPFs that although lacking chargeable amino acids can actively transport a substrate utilizing a proton gradient. These NPFs must have evolved an alternative proton-coupled mechanism that has not been characterized, yet.

Another question, not addressed in our study, is how these transporters evolved to be able to transport such a variety of substrates. So far no direct connection between the protein sequences and the substrate transported has been established, but as we learn more about this family of proteins we may in the future be able to predict which substrate each NPF can transport based on its sequence. More crystal structures are needed of NPFs bound to different substrates. In the meantime, structural modeling of NPFs can help identify important differences between NPFs belonging to different families and transporting different substrates.

In summary, our analysis of NPF sequences together with the available functional studies suggest that NPFs are versatile transporters. As symporters, they evolved from their initial role of proton-driven nitrate transporters to transport a variety of substrates, potentially using the same mechanism. As they expanded they acquired new roles: some evolved to passively facilitate nitrate or chloride efflux blurring the distinction between a transporter and a channel; others seem to have evolved to function as proton/potassium antiporters. This appears unique to NPFs.

## Conclusion

We observed that while many NPFs in plants have maintained elements or structural features that are at the basis of a conserved mechanism for proton-powered substrate transport, a significant number of NPFs lack such elements. In particular, we found that some plants contain NPFs that lack these elements and are passive nitrate or chloride efflux transporters. Other NPFs lack these elements but can still actively transport protons, suggesting the evolution of a different and still unknown mechanism for proton binding and transport. Our genome survey of plant NPFs combined with results from structural studies of NPF/POT transporters makes the case for extending the investigation to NPFs transporters from different subfamilies and plants. Since the function and mechanisms of NPFs cannot yet be easily inferred by sequence similarity, additional biochemical and structural studies as well as investigations of expression patterns and subcellular localization are needed to unravel the fascinating evolution of this family of plant proteins. Finally, although we did not address substrate recognition, we make the case for additional structural studies on NPFs in complex with the variety of different substrates that this family of proteins can transport.

## Author Contributions

AL collected and analyzed the NPF/PTR/POT/NRT1 sequences and crystal structures. NWM conducted the phylogenetic analysis. AL and RD wrote the manuscript. All authors read the manuscript and approved it.

## Conflict of Interest Statement

The authors declare that the research was conducted in the absence of any commercial or financial relationships that could be construed as a potential conflict of interest.

## Footnotes

1 https://phytozome.jgi.doe.gov/pz/portal.html

2 https://blast.ncbi.nlm.nih.gov/

3 https://www.peanutbase.org

4 http://itol.embl.de

5 http://weblogo.berkeley.edu/

6 https://swissmodel.expasy.org

## References

[B1] Almagro A. Lin S. H. Tsay Y. F. (2008). Characterization of the *Arabidopsis* nitrate transporter NRT1.6 reveals a role of nitrate in early embryo development. *Plant Cell* 20 3289–3299. 10.1105/tpc.107.056788 19050168 PMC2630450

[B2] Arnold K. Bordoli L. Kopp J. Schwede T. (2006). The SWISS-MODEL workspace: a web-based environment for protein structure homology modelling. *Bioinformatics* 22 195–201. 10.1093/bioinformatics/bti770 16301204

[B3] Biasini M. Bienert S. Waterhouse A. Arnold K. Studer G. Schmidt T. (2014). SWISS-MODEL: modelling protein tertiary and quaternary structure using evolutionary information. *Nucleic Acids Res.* 42 W252–W258. 10.1093/nar/gku340 24782522 PMC4086089

[B4] Boggavarapu R. Jeckelmann J. M. Harder D. Ucurum Z. Fotiadis D. (2015). Role of electrostatic interactions for ligand recognition and specificity of peptide transporters. *BMC Biol.* 6:58. 10.1186/s12915-015-0167-8 26246134 PMC4527243

[B5] Capella-Gutiérrez S. Silla-Martínez J. M. Gabaldón T. (2009). trimAl: a tool for automated alignment trimming in large-scale phylogenetic analyses. *Bioinformatics* 25 1972–1973. 10.1093/bioinformatics/btp348 19505945 PMC2712344

[B6] Chen C. Z. Lv X. F. Li J. Y. Yi H. Y. Gong J. M. (2012). Arabidopsis NRT1.5 is another essential component in the regulation of nitrate reallocation and stress tolerance. *Plant Physiol.* 159 1582–1590. 10.1104/pp.112.199257 22685171 PMC3425198

[B7] Corratgé-Faillie C. Lacombe B. (2017). Substrate (un)specificity of Arabidopsis NRT1/PTR family (NPF) proteins. *J. Exp. Bot.* 68 3107–3113. 10.1093/jxb/erw499 28186545

[B8] Crooks G. E. Hon G. Chandonia J. M. Brenner S. E. (2004). WebLogo: a sequence logo generator. *Genome Res.* 14 1188–1190. 10.1101/gr.849004 15173120 PMC419797

[B9] Doki S. Kato H. E. Solcan N. Iwaki M. Koyama M. Hattori M. (2013). Structural basis for dynamic mechanism of proton-coupled symport by the peptide transporter POT. *Proc. Natl. Acad. Sci. U.S.A.* 110 11343–11348. 10.1073/pnas.1301079110 23798427 PMC3710879

[B10] Donald J. E. Kulp D. W. DeGrado W. F. (2011). Salt bridges: geometrically specific, designable interactions. *Proteins* 79 898–915. 10.1002/prot.22927 21287621 PMC3069487

[B11] Drew D. Boudker O. (2016). Shared molecular mechanisms of membrane transporters. *Annu. Rev. Biochem.* 85 543–572. 10.1146/annurev-biochem-060815-014520 27023848

[B12] Fan S.-C. Lin C.-S. Hsu P.-K. Lin S.-H. Tsay Y.-F. (2009). The *Arabidopsis* nitrate transporter NRT1.7, expressed in phloem, is responsible for source-to-sink remobilization of nitrate. *Plant Cell* 21 2750–2761. 10.1105/tpc.109.067603 19734434 PMC2768937

[B13] Fan X. Xie D. Chen J. Lu H. Xu Y. Ma C. (2014). Over-expression of OsPTR6 in rice increased plant growth at different nitrogen supplies but decreased nitrogen use efficiency at high ammonium supply. *Plant Sci.* 227 1–11. 10.1016/j.plantsci.2014.05.013 25219300

[B14] Fang Z. Bai G. Huang W. Wang Z. Wang X. Zhang M. (2017). The rice peptide transporter *OsNPF7.3* is induced by organic nitrogen, and contributes to nitrogen allocation and grain yield. *Front. Plant Sci.* 8:1338. 10.3389/fpls.2017.01338 28824674 PMC5539172

[B15] Fowler P. W. Orwick-Rydmark M. Radestock S. Solcan N. Dijkman P. M. Lyons J. A. (2015). Gating topology of the proton-coupled oligopeptide symporters. *Structure* 23 290–301. 10.1016/j.str.2014.12.012 25651061 PMC4321885

[B16] Gojon A. Krouk G. Perrine-Walker F. Laugier E. (2011). Nitrate transceptor(s) in plants. *J. Exp. Bot.* 62 2299–2308. 10.1093/jxb/erq419 21239382

[B17] Guettou F. Quistgaard E. M. Raba M. Moberg P. Löw C. Nordlund P. (2014). Selectivity mechanism of a bacterial homolog of the human drug-peptide transporters PepT1 and PepT2. *Nat. Struct. Mol. Biol.* 21 728–731. 10.1038/nsmb.2860 25064511

[B18] Guettou F. Quistgaard E. M. Trésaugues L. Moberg P. Jegerschöld C. Zhu L. (2013). Structural insights into substrate recognition in proton-dependent oligopeptide transporters. *EMBO Rep.* 14 804–810. 10.1038/embor.2013.107 23867627 PMC3790050

[B19] Heckman D. S. Geiser D. M. Eidell B. R. Stauffer R. L. Kardos N. L. Hedges S. B. (2001). Molecular evidence for the early colonization of land by fungi and plants. *Science* 293 1129–1133. 10.1126/science.1061457 11498589

[B20] Ho C. H. Frommer W. B. (2014). Fluorescent sensors for activity and regulation of the nitrate transceptor CHL1/NRT1.1 and oligopeptide transporters. *eLife* 12:e01917. 10.7554/eLife.01917 24623305 PMC3950950

[B21] Ho C. H. Lin S. H. Hu H. C. Tsay Y. F. (2009). CHL1 functions as a nitrate sensor in plants. *Cell* 138 1184–1194. 10.1016/j.cell.2009.07.004 19766570

[B22] Hoang D. T. Chernomor O. von Haeseler A. Minh B. Q. Vinh L. S. (2018). UFBoot2: improving the ultrafast bootstrap approximation. *Mol. Biol. Evol.* 35 518–522. 10.1093/molbev/msx281 29077904 PMC5850222

[B23] Hu R. Qiu D. Chen Y. Miller A. J. Fan X. Pan X. (2016). Knock-Down of a tonoplast localized low-affinity nitrate transporter OsNPF7.2 affects rice growth under high nitrate supply. *Front. Plant Sci.* 7:1529. 10.3389/fpls.2016.01529 27826301 PMC5078692

[B24] Hu T. T. Pattyn P. Bakker E. G. Cao J. Cheng J. F. Clark R. M. (2011). The *Arabidopsis lyrata* genome sequence and the basis of rapid genome size change. *Nat. Genet.* 43 476–481. 10.1038/ng.807 21478890 PMC3083492

[B25] Huang W. Bai G. Wang J. Zhu W. Zeng Q. Lu K. (2018). Two splicing variants of OsNPF7.7 regulate shoot branching and nitrogen utilization efficiency in rice. *Front. Plant Sci.* 9:300. 10.3389/fpls.2018.00300 29568307 PMC5852072

[B26] Jørgensen M. E. Olsen C. E. Geiger D. Mirza O. Halkier B. A. Nour-Eldin H. H. (2015). A functional EXXEK motif is essential for proton coupling and active glucosinolate transport by NPF2.11. *Plant Cell Physiol.* 56 2340–2350. 10.1093/pcp/pcv145 26443378 PMC4675897

[B27] Kalyaanamoorthy S. Minh B. Q. Wong T. K. F. von Haeseler A. Jermiin L. S. (2017). ModelFinder: fast model selection for accurate phylogenetic estimates. *Nat. Methods* 14 587–589. 10.1038/nmeth.4285 28481363 PMC5453245

[B28] Krouk G. Lacombe B. Bielach A. Perrine-Walker F. Malinska K. Mounier E. (2010). Nitrate-regulated auxin transport by NRT1.1 defines a mechanism for nutrient sensing in plants. *Dev. Cell* 18 927–937. 10.1016/j.devcel.2010.05.008 20627075

[B29] Lee J. Sands Z. A. Biggin P. C. (2016). A numbering system for MFS transporter proteins. *Front. Mol. Biosci.* 3:21. 10.3389/fmolb.2016.00021 27314000 PMC4889909

[B30] Lee T. H. Kim J. Robertson J. S. Paterson A. H. (2017). Plant genome duplication database. *Methods Mol. Biol.* 1533 267–277. 10.1007/978-1-4939-6658-5-16 27987177

[B31] Lee T. H. Tang H. Wang X. Paterson A. H. (2012). PGDD: a database of gene and genome duplication in plants. *Nucleic Acids Res.* 41 D1152–D1158. 10.1093/nar/gks1104 23180799 PMC3531184

[B32] Léran S. Muños S. Brachet C. Tillard P. Gojon A. Lacombe B. (2013). Arabidopsis NRT1.1 is a bidirectional transporter involved in root-to-shoot nitrate translocation. *Mol. Plant* 6 1984–1987. 10.1093/mp/sst068 23645597

[B33] Léran S. Varala K. Boyer J. C. Chiurazzi M. Crawford N. Daniel-Vedele F. (2014). A unified nomenclature of nitrate transporter 1/PEPTIDE transporter family members in plants. *Trends Plant Sci.* 19 5–9. 10.1016/j.tplants.2013.08.008 24055139

[B34] Letunic I. Bork P. (2016). Interactive tree of life (iTOL) v3: an online tool for the display and annotation of phylogenetic and other trees. *Nucleic Acids Res.* 44 W242–W245. 10.1093/nar/gkw290 27095192 PMC4987883

[B35] Li B. Byrt C. Qiu J. Baumann U. Hrmova M. Evrard A. (2016). Identification of a stelar-localized transport protein that facilitates root-to-shoot transfer of chloride in arabidopsis. *Plant Physiol.* 170 1014–1029. 10.1104/pp.15.01163 26662602 PMC4734554

[B36] Li B. Qiu J. Jayakannan M. Xu B. Li Y. Mayo G. M. (2017). AtNPF2.5 Modulates Chloride (Cl-) Efflux from Roots of *Arabidopsis thaliana*. *Front. Plant Sci.* 5:2013. 10.3389/fpls.2016.02013 28111585 PMC5216686

[B37] Li H. Yu M. Du X. Q. Wang Z. F. Wu W. H. Quintero F. J. (2017). NRT1.5/NPF7.3 functions as a proton-coupled H(+)/K(+) antiporter for K(+) loading into the xylem in arabidopsis. *Plant Cell* 29 2016–2026. 10.1105/tpc.16.00972 28739644 PMC5590498

[B38] Li J. Y. Fu Y. L. Pike S. M. Bao J. Tian W. Zhang Y. (2010). The arabidopsis nitrate transporter NRT1.8 functions in nitrate removal from the xylem sap and mediates cadmium tolerance. *Plant Cell* 22 1633–1646. 10.1105/tpc.110.075242 20501909 PMC2899866

[B39] Lin S. H. Kuo H. F. Canivenc G. Lin C. S. Lepetit M. Hsu P. K. (2008). Mutation of the Arabidopsis NRT1.5 nitrate transporter causes defective root-to-shoot nitrate transport. *Plant Cell* 20 2514–2528. 10.1105/tpc.108.060244 18780802 PMC2570733

[B40] Lomize M. A. Pogozheva I. D. Joo H. Mosberg H. I. Lomize A. L. (2012). OPM database and PPM web server: resources for positioning of proteins in membranes. *Nucleic Acids Res.* 40 D370–D376. 10.1093/nar/gkr703 21890895 PMC3245162

[B41] Lyons J. A. Parker J. L. Solcan N. Brinth A. Li D. Shah S. T. (2014). Structural basis for polyspecificity in the POT family of proton-coupled oligopeptide transporters. *EMBO Rep.* 15 886–893. 10.15252/embr.201338403 24916388 PMC4149780

[B42] McWilliam H. Li W. Uludag M. Squizzato S. Park Y. M. Buso N. (2013). Analysis tool web services from the EMBL-EBI. *Nucleic Acids Res.* 41 W597–W600. 10.1093/nar/gkt376 23671338 PMC3692137

[B43] Miller M. A. Pfeiffer W. Schwartz T. (2010). “Creating the CIPRES science gateway for inference of large phylogenetic trees,” in *Proceedings of the Gateway Computing Environments Workshop (GCE)* (New Orleans, LA: IEEE). 10.1109/GCE.2010.5676129

[B44] Newstead S. (2011). Towards a structural understanding of drug and peptide transport within the proton-dependent oligopeptide transporter (POT) family. *Biochem. Soc. Trans.* 39 1353–1358. 10.1042/BST0391353 21936814

[B45] Newstead S. (2015). Molecular insights into proton coupled peptide transport in the PTR family of oligopeptide transporters. *Biochim. Biophys. Acta* 1850 488–499. 10.1016/j.bbagen.2014.05.011 24859687 PMC4331665

[B46] Newstead S. (2017). Recent advances in understanding proton coupled peptide transport via the POT family. *Curr. Opin. Struct. Biol.* 45 17–24. 10.1016/j.sbi.2016.10.018 27865112 PMC5628733

[B47] Newstead S. Drew D. Cameron A. D. Postis V. L. Xia X. Fowler P. W. (2011). Crystal structure of a prokaryotic homologue of the mammalian oligopeptide-proton symporters, PepT1 and PepT2. *EMBO J.* 30 417–426. 10.1038/emboj.2010.309 21131908 PMC3025455

[B48] Nour-Eldin H. H. Andersen T. G. Burow M. Madsen S. R. Jørgensen M. E. Olsen C. E. (2012). NRT/PTR transporters are essential for translocation of glucosinolate defence compounds to seeds. *Nature* 488 531–534. 10.1038/nature11285 22864417

[B49] Ouyang J. Cai Z. Xia K. Wang Y. Duan J. Zhang M. (2010). Identification and analysis of eight peptide transporter homologs in rice. *Plant Sci.* 179 374–382. 10.1016/j.plantsci.2010.06.013

[B50] Panchy N. Lehti-Shiu M. Shiu S. H. (2016). Evolution of gene duplication in plants. *Plant Physiol.* 171 2294–2316. 10.1104/pp.16.00523 27288366 PMC4972278

[B51] Parker J. L. Li C. Brinth A. Wang Z. Vogeley L. Solcan N. (2017). Proton movement and coupling in the POT family of peptide transporters. *Proc. Natl. Acad. Sci. U.S.A.* 114 13182–13187. 10.1073/pnas.1710727114 29180426 PMC5740623

[B52] Parker J. L. Newstead S. (2014). Molecular basis of nitrate uptake by the plant nitrate transporter NRT1.1. *Nature* 507 68–72. 10.1038/nature13074 24572366 PMC3982047

[B53] Paulsen I. T. Skurray R. A. (1994). The POT family of transport proteins. *Trends Biochem. Sci.* 19:404 10.1016/0968-0004(94)90087-6 7817396

[B54] Pellizzaro A. Alibert B. Planchet E. Limami A. M. Morère-Le Paven M. C. (2017). Nitrate transporters: an overview in legumes. *Planta* 246 585–595. 10.1007/s00425-017-2724-6 28653185

[B55] Quistgaard E. M. Martinez Molledo M. Löw C. (2017). Structure determination of a major facilitator peptide transporter: inward facing PepTSt from Streptococcus thermophilus crystallized in space group P3121. *PLoS One* 12:e0173126. 10.1371/journal.pone.0173126 28264013 PMC5338821

[B56] Saier M. H. Jr. Reddy V. S. Tsu B. V. Ahmed M. S. Li C. Moreno-Hagelsieb G. (2016). The transporter classification database (TCDB): recent advances. *Nucleic Acids Res.* 44 D372–D379. 10.1093/nar/gkv1103 26546518 PMC4702804

[B57] Sanz-Luque E. Chamizo-Ampudia A. Llamas A. Galvan A. Fernandez E. (2015). Understanding nitrate assimilation and its regulation in microalgae. *Front. Plant Sci.* 6:899. 10.3389/fpls.2015.00899 26579149 PMC4620153

[B58] Segonzac C. Boyer J. C. Ipotesi E. Szponarski W. Tillard P. Touraine B. (2007). Nitrate efflux at the root plasma membrane: identification of an Arabidopsis excretion transporter. *Plant Cell* 19 3760–3777. 10.1105/tpc.106.048173 17993627 PMC2174868

[B59] Sievers F. Wilm A. Dineen D. Gibson T. J. Karplus K. Li W. (2011). Fast, scalable generation of high-quality protein multiple sequence alignments using clustal omega. *Mol. Syst. Biol.* 7:539. 10.1038/msb.2011.75 21988835 PMC3261699

[B60] Solcan N. Kwok J. Fowler P. W. Cameron A. D. Drew D. Iwata S. (2012). Alternating access mechanism in the POT family of oligopeptide transporters. *EMBO J.* 31 3411–3421. 10.1038/emboj.2012.157 22659829 PMC3419923

[B61] Steiner H. Y. Naider F. Becker J. M. (1995). The PTR family: a new group of peptide transporters. *Mol. Microbiol.* 16 825–834. 10.1111/j.1365-2958.1995.tb02310.x 7476181

[B62] Sun J. Bankston J. R. Payandeh J. Hinds T. R. Zagotta W. N. Zheng N. (2014). Crystal structure of the plant dual-affinity nitrate transporter NRT1.1. *Nature* 507 73–77. 10.1038/nature13074 24572362 PMC3968801

[B63] Taochy C. Gaillard I. Ipotesi E. Oomen R. Leonhardt N. Zimmermann S. (2015). The Arabidopsis root stele transporter NPF2.3 contributes to nitrate translocation to shoots under salt stress. *Plant J.* 83 466–479. 10.1111/tpj.12901 26058834

[B64] Terada T. Inui K. (2012). Recent advances in structural biology of peptide transporters. *Curr. Top. Membr.* 70 257–274. 10.1016/B978-0-12-394316-3.00008-9 23177989

[B65] Trifinopoulos J. Nguyen L. T. von Haeseler A. Minh B. Q. (2016). W-IQ-TREE: a fast online phylogenetic tool for maximum likelihood analysis. *Nucleic Acids Res.* 44 W232–W235. 10.1093/nar/gkw256 27084950 PMC4987875

[B66] Tsay Y. F. Chiu C. C. Tsai C. B. Ho C. H. Hsu P. K. (2007). Nitrate transporters and peptide transporters. *FEBS Lett.* 581 2290–2300. 10.1016/j.febslet.2007.04.047 17481610

[B67] Tsay Y. F. Schroeder J. I. Feldmann K. A. Crawford N. M. (1993). The herbicide sensitivity gene CHL1 of arabidopsis encodes a nitrate-inducible nitrate transporter. *Cell* 72 705–713. 10.1016/0092-8674(93)90399-B 8453665

[B68] von Wittgenstein N. J. Le C. H. Hawkins B. J. Ehlting J. (2014). Evolutionary classification of ammonium, nitrate, and peptide transporters in land plants. *BMC Evol. Biol.* 20:11. 10.1186/1471-2148-14-11 24438197 PMC3922906

[B69] Wang J. Lu K. Nie H. Zeng Q. Wu B. Qian J. (2018). Rice nitrate transporter OsNPF7.2 positively regulates tiller number and grain yield. *Rice* 11:12. 10.1186/s12284-018-0205-6 29484500 PMC5826914

[B70] Wang Y. Y. Tsay Y. F. (2011). *Arabidopsis* nitrate transporter NRT1.9 is important in phloem nitrate transport. *Plant Cell* 23 1945–1957. 10.1105/tpc.111.083618 21571952 PMC3123939

[B71] Yan N. (2013). Structural advances for the major facilitator superfamily (MFS) transporters. *Trends Biochem. Sci.* 38 151–159. 10.1016/j.tibs.2013.01.003 23403214

[B72] Yan N. (2015). Structural biology of the major facilitator superfamily transporters. *Annu. Rev. Biophys.* 44 257–283. 10.1146/annurev-biophys-060414-0-33901 26098515

[B73] Zhang X. C. Zhao Y. Heng J. Jiang D. (2015). Energy coupling mechanisms of MFS transporters. *Protein Sci.* 24 1560–1579. 10.1002/pro.2759 26234418 PMC4594656

[B74] Zhao Y. Mao G. Liu M. Zhang L. Wansg X. Zhang X. C. (2014). Crystal structure of the E. coli peptide transporter YbgH. *Structure* 22 152–160. 10.1016/j.str.2014.06.008 25066136

