# Supplementary material for: Genome Mining of Plant NPFs Reveals Varying Conservation of Signature Motifs Associated With the Mechanism of Transport
Source: Front Plant Sci. 2018 Dec 4;9:1668. doi: 10.3389/fpls.2018.01668 (PMC6288477; doi:10.3389/fpls.2018.01668)
Supplement: Supplementary file 3 [file Table_3.docx]

**SUPPLEMENTARY** **TABLE 3.**

**List of putative NPF2a/NAXT proteins from 39 fully sequenced plant genomes.**

Based on multiple sequence alignment with NPF2.7/NAXT, the first functionally characterized protein from the NPF2 subfamily, we identified a total of 94 putative NAXT proteins from dicots and monocots plants. These NPFs form a distinct subclade in the phylogenetic tree that we named NPF2a (Figure 5A). All TMH1 from the NPF2a subclade lack chargeable amino acids in the ExxER/K motif region, as evident by comparing with the AtNPF6.3 TMH1 sequence (sequence at the top with chargeable amino acid in bold).

| **Dicots** | **NPF** | **TMH1 Sequence** |
| --- | --- | --- |
| ***Arabidopsis thaliana*** | **NPF6.3** | GGWASAAMILCI**E**AV**ER**LTTLGIGVNLVTYLTGTM |
| *Aquilegia coerulea* | NPF2.1  NPF2.2  NPF2.3 | GGWTTFPFVTVSLLGLSIAVAGWMTNLIVYLIQKF  GGWTTFPFITVSALGLSIAAAGWTTNLIVYLIQKF  GGWITFPFITVALTGLTIAASGWMTNLIVYLIEQF |
| *Arabidopsis thaliana* | NPF2.1  NPF2.2  NPF2.3  NPF2.4  NPF2.5  NPF2.6  NPF2.7 | GGCITFPFMIATLLGISVTSYGWVLNLIVFLIEEY  GGWKTFPFMIATLLGLSIASFGWVMNLVVFLIKEF  GGWITFPFMLATLLGLSVTSFGWVMNLIVFLIEEF  GGWITLPFMLVTLLGMSITYFGWVMNLIVFLIEEF  GGWITLPFMLVTLLGMSITSFGWGMNLIVFLIEEF  GGRITFPFMIVTLFGLTLATLGWLQNLIVYLIEEY  GGWITFPFMIATLLGLTIAAWGWLLNLIVYLIEEF |
| *Arabidopsis lyrata* | NPF2.2  NPF2.3  NPF2.4  NPF2.6 | GGWKTFPFMIATLLGLSIASFGWVMNLVVFLIDVF  GGWITFPFMIATLLGLSVTSFGWVMNLIVFLIEEF  GGWTTLPFMLVTLLGMSITSFGWGMNLIVFLIEEF  GGWITFPFMIVTLLGLTIAAWGWLLNLIVYLIEEF |
| *Arachis duranensis* | NPF2.1 | GGWPSFPFIIATVAGITLASSGIWANLIVYLIQEF |
| *Brassica rapa* | NPF2.1  NPF2.2  NPF2.3  NPF2.4 | GGWITFPFMMATLLGMSITSFGWVLNLIVFLIEEF  GGWITLPFMMATLLGMSITSFGWVLNLIVFLIEEF  GGWITFPFMIATLLGLTIAAWGWLLNLIVYLIEEF  GGWITFPFMIATLLFLSLAVLGWLFNLIVFLIEEF |
| *Capsella rubella* | NPF2.8  NPF2.10  NPF2.11 | GGWITFPFMIATLLGLTIAAWGWLLNLIVYLIEEF  GGWITFPFMIATLLGLSVTSFGWVMNLIVFLIEEF  GGWITFPFLIATLLGLSIASFGWIMNLVVFLIKEF |
| *Carica papaya* | NPF2.1  NPF2.3  NPF2.4  NPF2.5 | GGWVTFPFMAGTLMGLTIAMGGWATSLVVYMIEKF  GGWITFPFITGALAGLTLCSGGWIGNLIVYLIQEF  GGWITFPFIVGAVAGSALCGAGWISNLIVYLIQEF  SDWITFPFIVGAVAGSTLCGAGWVSNLIVYLIQEF |
| *Cicer arietinum* | NPF2.10 | GGWISFSFIIGIVTGSSLASTAIIGNLIVYLIREF |
| *Cucumis sativus* | NPF2.1  NPF2.3  NPF2.4 | GGWITFTFIIGTFACMTLATGGWLSNLIVYLIKEY  GGWITFPFVIGTFACMTLATGGWLSNLIVYLIKEY  GGWITFPFIIGCLGCMTLTAGGFLANLIVYLIKEY |
| *Daucus carota* | NPF2.6  NPF2.7  NPF2.8 | GGWITFPFIIATMGGLSLACGGWTMNLMVYLIEEF  GGWITFPFIIASTAALSLAAGGWMYNLMVYLIQEF  GGWKAFPYIIGS**E**MGLSLASTGWGCNLTVYLITVF |
| *Eucaliptus grandis* | NPF2.1  NPF2.2  NPF2.3 | AGWNTFPFIIGAMALLMLAGTGWMANLIVYLIQEF PGWITFPFIIGSAALWTMGGTGWMTNFIVYLIKEF AGWATFPFITGAAALLSVGATGWMTNLIVYLIKEF |
| *Eutrema salsugineum* | NPF2.1  NPF2.2 | GGWITFPFMIATLLGLSITSFGWVMNLIVFLIEEF  GGWITFPFMIATLLGLTVAAWGWLLNLIVYLIEEF |
| *Fragaria vesca* | NPF2.1  NPF2.2  NPF2.3  NPF2.4  NPF2.6  NPF2.7  NPF2.8 | GSWTTFPFVIATVLGLTVAAGGWGANLIVFLITKF  GGWTTFPFVIATVLGLSVAGGGWASNLIVFLITKF  GGWTTFPFVIATVLGLSLAAGGWASNLIVFLITKF  GGWITFPFIIGATLGLTLAVGGWLSNLIVFLIQEF  GGWITFPFTIGALLGLTFAVGGWLSNLLVFLVQEF  GGWITFPFITGALAGLTVASGGWGSNLIVYLIQEF  GGWITFPFITGALAGLTLAAGGWASNIIVYLIQEF |
| *Glycine max* | NPF2.1 | GGWISFPFTIGSTAGISVASAGIIGNLIVYLIREF |
| *Gossypium raimondii* | NPF2.1  NPF2.2  NPF2.3  NPF2.4 | GGWFTFFFVSATLTGLMIAGWGWLTNLIVYLIEEF  GGFITLFFISGTLSGVMLSGFGWLANLIVYLVQEF  GGWATFPFIAGALIGSSIAASGWANNLIVYMIEKF  GGWVTFPFITGAVLGLGLAGTGWMANLIIYLIQQF |
| *Lotus japonicus* | NPF2.6 | GGWRSFPFIIGAATGSSLASAGIIANLIVYLIQEF |
| *Manihot esculenta* | NPF2.1 | GSWVTFPFIIGTFSCLTLAGAGWLFNIIVYLIKEF |
| *Medicago truncatula* | NPF2.2  NPF2.4  NPF2.5 | GGWISFPFFIGMVAGLSLASAGIGGNLIVYLIKEF  GGWISFPFFIGMITGLSLASTGIVGNLIVYLISEF  GGWISSPFFIGMIAGMSLGSTGIVGNLIVYLTREF |
| *Mimulus guttatus* | NPF2.6 | GGWTTFPFIIGTMGCLTLAAGGWVANLIVYLIEEF |
| *Populus trichocarpa* | NPF2.1 | GGWTTFPFIIGAVMGLTLAAGAGSANLIVFLVTVM |
| *Prunus persica* | NPF2.1  NPF2.2  NPF2.3  NPF2.4  NPF2.6  NPF2.7 | GGWIIYFFIIGTLMCLTLAAGGWQSNLIVYLIQEY  GGWITFPFVTGSMLGLSIAGGGWGSNLIVFLITKF  GGWKTFPFISGSVLGLSVAAGGWASNLTVFLITKF  GGWITFPFITGALAGLTLAAGGWLSNLIVYLIQEF  GGWITFPFITGALVGLTLTAGGWLSNLIVFLTEEF  GGWITFPFITGALVGLTLTAGGWLSNLIVFLIEEF |
| *Ricinus communis* | NPF2.4  NPF2.7  NPF2.8 | GNWITFPFIIGTMAGVTLAGGGYLANLIVYLIEEF GKWITFPFVTGTMVGLTLAGVGYLSNIIVYLIEEF  ANWITFPFVTGTMVGLTLAGVGYLSNIIVHLIEEF |
| *Solanum lycopersicum* | NPF2.1  NPF2.2  NPF2.10  NPF2.11  NPF2.12  NPF2.13  NPF2.14 NPF2.16 | GGWISFPFIIGSMAGLSLAAAGWNNNLIVYLIEEF  GGWITFPFIIATTAGLNIAAAGWMNNLTVYLIEEF  GGWITFPFIIATMAGLSLASGGWTSNLIVYLIDEF  GGWITFPFIIATMAGLSLAAGGWTSNLIVFLINEF  GGWITFPFIIATTTCLTLAFGGWTSNLIVYLIKEF  GGWITFPFIIASSVGFTLAFGGLTSNLIVYLIKEF  GGWITFPFIIASSVGLTLAFGGWTSNLIVYLIKEF  GGWITFPFIIATTVGLALSFVGWTSNLIVYLIKEF |
| *Solanum tuberosum* | NPF2.2  NPF2.3  NPF2.11  NPF2.12  NPF2.13 | GGWITFPFIIATMAGLSLAAGGWTSNLIVFLINEF  GGWITFPFTIATTAGLNIAAAGWMNNLTVYLIEEF  GGWISFPFIIGSMAGLSLAAAGWNNNLIVYLIEEF  GGWITFPFIIATMAGLSLASGGWTSNLIVYLINEF  GGWITFPFIIATSTCLTLAFGGWTSNLIVYLIKEF |
| *Theobroma cacao* | NPF2.1  NPF2.3  NPF2.5  NPF2.7 | GGWITFFFVSATLTGLMIAGWGWLTNLIVYLIEEF  GGWTTFPLIAGTLMGSSIAVSGWANSLIVYMIEKF  GGWTTFPFIIGSMTGLSLVAGGWGANLIVFLINEF  GGWITFPFIIGTLAGLTLAGGGWVANLIVYLIQEF |
| *Vitis vinifera* | NPF2.1  NPF2.2 | GGWITFLFIAGALTGLTLAAGGWGANLIVYLIEEF GGWITFLFIAGALTGLTLAAGGWGANLIVYLIEEF |
| **Monocots** |  |  |
| *Ananas comosus* | NPF2.4 | GGWITFPFIAGSVFGLGLAISGAMSNFIVYLIKEY |
| *Brachypodium distachyon* | NPF2.1 | GGWITFPFLAVAILGLGLATGGALSNMVVYLIKEY |
| *Musa acuminata* | NPF2.9  NPF2.10 | GGWITFPFILVSNLGLGLALSGATANLIVYLVEEY  GGWITVPFIIGSMLGQGLALTGAMGNLVVYLIKEY |
| *Oryza sativa* | NPF2.1 | GGWITFPFMAVSLLAFGLSSAGAMGNLVVYLVKEY |
| *Setaria italica* | NPF2.1  NPF2.2  NPF2.3  NPF2.4 | GGWITLPFLAGSVIGLGMAMSATSSNLIVYLIRKY  GGWITLPFIAGSMLGLGLAINGTTSNLLVYLLKEY  GGWITFPFLAVAMLGLGVARGGATSNFVVYLVKKY  GGWITFPFLGVAMMGLGVATSGALNNLVVYLIKEY |
| *Sorghum bicolor* | NPF2.9 | GGWITFPFLGAAMMGLGVAMSGVLSNLVVYLIKEY |
