# Supplementary material for: Genome Mining of Plant NPFs Reveals Varying Conservation of Signature Motifs Associated With the Mechanism of Transport
Source: Front Plant Sci. 2018 Dec 4;9:1668. doi: 10.3389/fpls.2018.01668 (PMC6288477; doi:10.3389/fpls.2018.01668)
Supplement: Supplementary file 4 [file Data_Sheet_1.pdf]

SUPPLEMENTARY FIGURE 1.

Expanded view of Figure 5. When this image is enlarged to 2400%, it is possible to see the identities of the individual NPF transporters in the phylogeny. NPFs from *A. thaliana* are labeled with red font.

SUPPLEMENTARY FIGURE 2.

Phylogenetic tree of nitrate transporters from 42 plants. Normal view of the maximum-likehood phylogenetic tree of 2383 NPFs sequences from 42 plants visualized with iTOL (Letunic and Bork, 2016). Tree branches are colored consistently with Figure 5 and Supplementary Figure 1. Transporter color is based on the eight subfamilies proposed in Léran et al. (2014). The two subclades that correlate with specific characteristics of the ExxER/K motif, NPF2a and NPF7a, are in light orange and light blue, respectively. Percent bootstrap values from 2000 replicates are given for each branch of the phylogenetic tree. A text label near each NPF contains the corresponding ExxER/K motif sequence. Amino acids are colored based on their chemical properties: hydrophobic amino acids are grey, polar are green, basic are blue, and acidic are red.

SUPPLEMENTARY FIGURE 3.

Phylogenetic tree of nitrate transporters in green algae.

A phylogenetic tree was obtained after aligning green algae NRT1 sequences with our collection of plant NPFs. The algal NRT1s (*green lines*) align with NPFs from mosses (*blue lines*). All algal NRT1s and the moss NPFs have a conserved ExxER motif and can form the TMH4-TMH10 salt bridge. On the contrary, only NRT1s from *B. prasinos*, *M. pusilla*, *O. lucimarinus* and *O. Tauris* which are Mamiellophyceae, and two NPFs from P. patens, PpatNPF8.5 and PpatNPF8.6, have residues that can form the TMH1-TMH7 salt bridge.

SUPPLEMENTARY TABLE 1.

Identification of NPF/POT/NRT genes in plants, algae, bacteria, and eukaryotes.

SUPPLEMENTARY TABLE 2.

Distribution of NPF genes in the eight subfamilies and conservation of the ExxER/K motif within genomes and subfamilies. The number of NPFs belonging to each of the eight subfamilies is shown for each genome. A green color scale was used to represent the abundance of NPFs, with darker green cells representing subfamilies or genomes with more NPFs. The conservation of the ExxER/K motif is shown for each subfamily and each genome. A red color scale was used to represent the conservation of the motif, with darker red cells representing the subfamilies or genomes with more conserved motif.
