# Supplementary figures and images for: Genome Mining of Plant NPFs Reveals Varying Conservation of Signature Motifs Associated With the Mechanism of Transport
Source: Front Plant Sci. 2018 Dec 4;9:1668. doi: 10.3389/fpls.2018.01668 (PMC6288477; doi:10.3389/fpls.2018.01668)

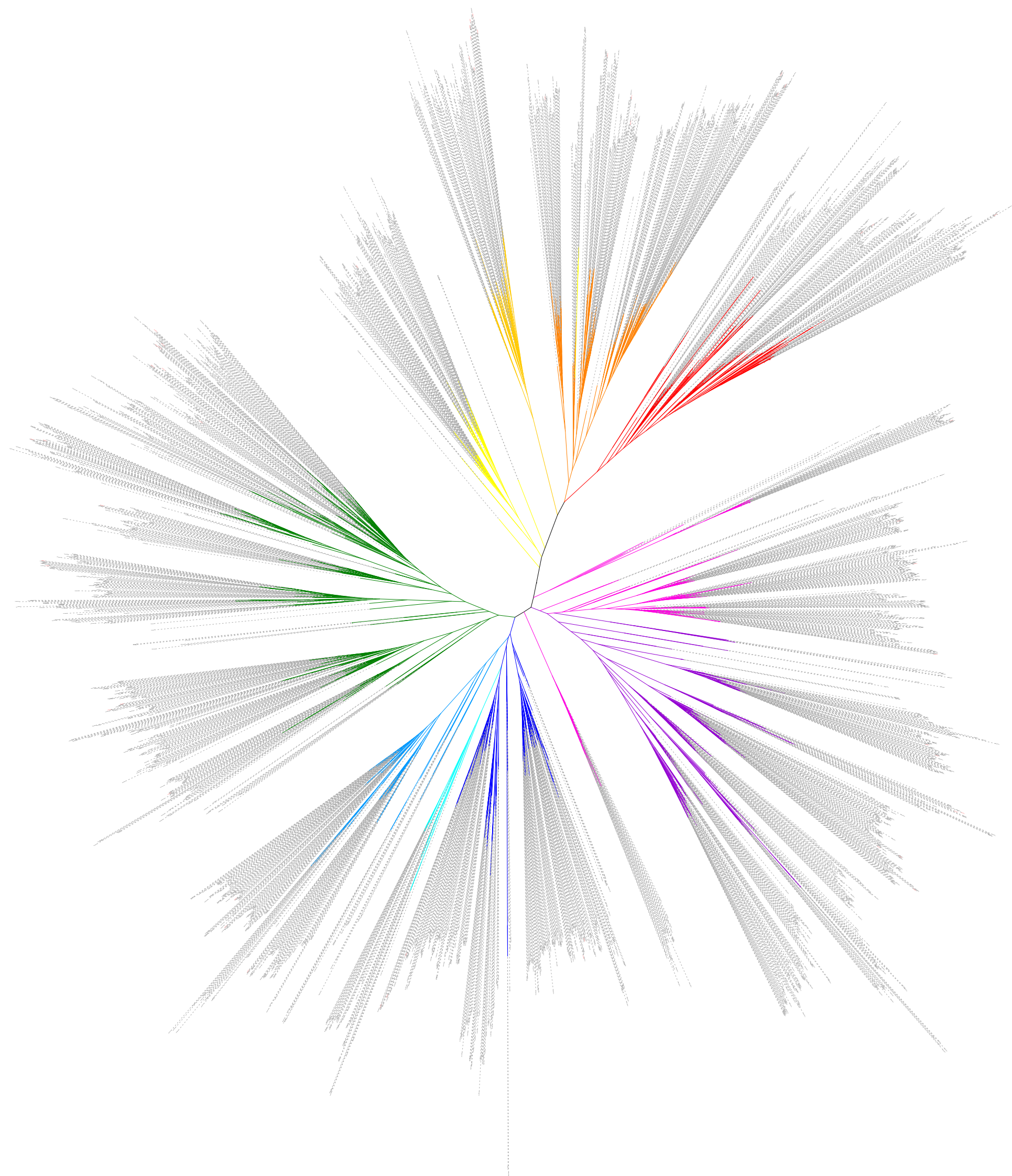

Supplement: Supplementary file 5 [file Image_1.pdf]

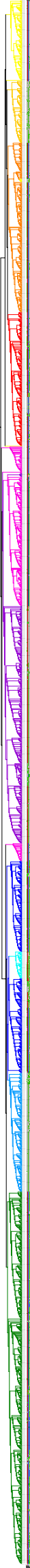

Supplement: Supplementary file 6 [file Image_2.pdf]

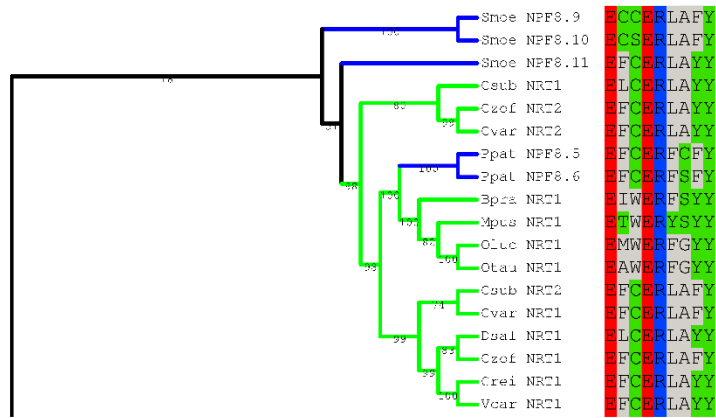

Supplement: Supplementary file 7 [file Image_3.pdf]
